# Transcriptome Profiling of Wild-Type and *pga*-Knockout Mutant Strains Reveal the Role of Exopolysaccharide in *Aggregatibacter actinomycetemcomitans*

**DOI:** 10.1371/journal.pone.0134285

**Published:** 2015-07-29

**Authors:** Mayilvahanan Shanmugam, Faiha El Abbar, Narayanan Ramasubbu

**Affiliations:** Department of Oral Biology, Rutgers School of Dental Medicine, Newark, New Jersey, United States of America; Loyola University Chicago, UNITED STATES

## Abstract

Exopolysaccharides have a diverse set of functions in most bacteria including a mechanistic role in protecting bacteria against environmental stresses. Among the many functions attributed to the exopolysaccharides, biofilm formation, antibiotic resistance, immune evasion and colonization have been studied most extensively. The exopolysaccharide produced by many Gram positive as well as Gram negative bacteria including the oral pathogen *Aggregatibacter actinomycetemcomitans* is the homopolymer of β(1,6)-linked N-acetylglucosamine. Recently, we reported that the PGA-deficient mutant of *A*. *actinomycetemcomitans* failed to colonize or induce bone resorption in a rat model of periodontal disease, and the colonization genes, *apiA* and *aae*, were significantly down regulated in the mutant strain. To understand the role of exopolysaccharide and the *pga* locus in the global expression of *A*. *actinomycetemcomitans*, we have used comparative transcriptome profiling to identify differentially expressed genes in the wild-type strain in relation to the PGA-deficient strain. Transcriptome analysis revealed that about 50% of the genes are differently expressed (P < 0.05 and fold change >1.5). Our study demonstrated that the absence of the *pga* locus affects the genes involved in peptidoglycan recycling, glycogen storage, and virulence. Further, using confocal microscopy and plating assays, we show that the viability of *pga* mutant strain is significantly reduced during biofilm growth. Thus, this study highlights the importance of *pga* genes and the exopolysaccharide in the virulence of *A*. *actinomycetemcomitans*.

## Introduction

Bacteria find themselves in diverse environmental conditions and as a necessity to cope with those of stress, resort to a surface-associated biofilm state. Oral bacteria are no exception to this fundamental survival mode. To remain under harsh conditions of limited nutrients, many oral bacteria exist within the biofilm as aggregated clusters that are encased inside a self-produced extracellular matrix. This complex matrix varies in its composition of proteins, nucleic acids and exopolysaccharide (EPS), which are bound to the bacterial cell surface. Among the oral bacteria, *Aggregatibacter actinomycetemcomitans*, an oral pathogen implicated as a causative agent for localized aggressive periodontitis, produces EPS comprised of a homopolysaccharide made up of N-acetylglucosamine with β(1–6) glycosidic linkage (PGA) [1–3]. This polysaccharide has been shown to be important in colonization [4], aggregation/adhesion [5], and resistance to killing by phagocytes [6]. Several other pathogens including *Actinobacillus pleuropneumoniae*, *Escherichia coli* [7], *Yersinia pestis*, *Bordetella* spp. [8], *Staphylococcus aureus* and *S*. *epidermidis* [9] also produce structurally similar PGA. In these bacteria, PGA has been ascribed to functions such as surface attachment, intercellular adhesion, biofilm formation, epithelial cell attachment, and resistance to killing by antibiotics, antimicrobial peptides and human PMNs. Another potential function suggested for PGA is its mechanistic role in protecting bacteria against environmental stresses.

Stress conditions for bacteria in a biofilm are due to many factors including limited nutrients in the environment. Since cells are at risk under stress, rapid adaptation is crucial for cell survival. Although EPS displays diversity in its structure and composition across species, most bacteria have adapted to synthesize EPS under different growth conditions. In general, microbes activate the machinery for EPS synthesis by channeling limited resources to its production, leading to accumulation in stationary phase [10]. To define the mechanisms and molecular components activated during stress responses, transcriptome analyses have been utilized [11]. Often organisms respond to different stresses by regulating gene expression of universal stress proteins, heat/cold shock proteins, altering the fatty acid composition and accumulating fatty acids to maintain membrane fluidity [12, 13]. Based on how severe the stress is, bacteria might undergo changes that affect their dispersal and the extent of cellular protein damage. Ultimately these changes may lead to death [14].

Although many oral bacteria including *A*. *actinomycetemcomitans* produce EPS, the advantages for EPS-producing bacteria are still underexplored with respect to how they respond to stress and nutrient starvation. If cells tend to aggregate in biofilm state, as has been shown for *A*. *actinomycetemcomitans* [15, 16], EPS can aid in surface colonization and affect colony morphology. It is of particular interest to note that unlike some bacteria, *A*. *actinomycetemcomitans* has only one type of EPS (PGA) other than the capsular polysaccharide. Bacteria such as *Pseudomonas putida*, on the other hand, produce alginate as the main EPS but also produce cellulose (bcs), putida exopolysaccharide a (pea), and putida exopolysaccharide b (peb) [14]. In the absence of alginate other tolerance mechanisms are activated in *P*. *putida* to maintain homeostasis. Since *A*. *actinomycetemcomitans* produces only PGA as its EPS, it would be interesting to determine the various cellular responses in the PGA-negative background and assess how the absence of PGA would be tolerated by *A*. *actinomycetemcomitans*. Using a murine abscess model, previous transcriptome studies have shown that *in vivo* growth of *A*. *actinomycetemcomitans* significantly modifies its energy production and conversion [17]. A comparative analysis of wild-type strain (IDH781) and a *pga* knock-out strain during biofilm growth will provide considerable information for the importance of PGA for *A*. *actinomycetemcomitans*.

We have previously generated a *pga* knockout strain (EA1002) lacking the PGA synthesis operon *pgaABCD* in *A*. *actinomycetemcomitans* [16]. In a recent study, we demonstrated that EA1002 failed to induce bone resorption in a rat model for periodontitis [4]. In addition, we also showed that in the early stages of *in vitro* biofilm growth of EA1002, the expression levels of virulence genes that are involved in colonization such as *flp-1*, *apiA* and *aae* are down-regulated.

To further understand the gene expression changes that render EA1002 strain ineffective in colonization and bone resorption, in this report, we have used comparative transcriptome profiling to identify genes that are differentially expressed in EA1002. While examining the data, we asked the following questions: 1) Are there specific cellular processes that are affected or shifted in EA1002; 2) Are there differentially expressed genes that will point towards observable physiologic changes between IDH781 and EA1002; and 3) What metabolic pathways, if any, are affected in EA1002? As shown below, in this report, we provide data showing how the absence of PGA synthesis genes in EA1002 affects various cellular processes culminating in a metabolic shift from aerobic to anaerobic growth for energy production. Also the EA1002 cells undergo a change in glycogen accumulation and near complete cell death in the biofilm state compared to IDH781. This study highlights the importance of *pga* genes, which could be suitable targets for therapeutic intervention for *A*. *actinomycetemcomitans* and possibly other bacteria that produce PGA.

## Materials and Methods

### Bacterial strains and growth conditions

*A*. *actinomycetemcomitans* strain IDH781 (serotype d), a rough phenotype clinical isolate and the isogenic pgaABCD deletion mutant (EA1002), a rough phenotype derivative [16] were grown on trypticase soy agar (TSA) plates containing yeast extract (0.6%), sodium bicarbonate (0.4%) and glucose (0.75%) for 2 days in a 37°C incubator at 10% CO_2_/90% air atmosphere. Single colonies were scraped from the well-separated area from the agar plates, suspended in Tryptic soy broth (TSB) containing yeast extract (0.6%), sodium bicarbonate (0.4%) and glucose (0.75%). The aggregated cells were dispersed with hand held homogenizer (Cat. No. 749540–0000, Kimble Chase, Vineland, NJ) and the non-aggregated cells were removed by allowing the culture to incubate on ice for two minutes, and the cells in the top portion of the culture were collected. The cell density was adjusted to ~10^8^ cells per ml (OD_600_ = 0.80). Inocula from this culture were used for seeding subsequent experiments, *in vitro* biofilm growth and cell harvest (100 μl for 10 ml of TSB medium; see above)). Ten ml of inoculated medium was plated on 10 cm polystyrene plates and incubated at 10% CO_2_/90% air atmosphere. The supernatant was removed after 16 h, plates were washed with ice cold PBS and the biofilm RNA was stabilized by adding ice cold 0.9% saline supplemented with 1/10^th^ volume of citric acid saturated phenol mixture in 95% ethanol. The attached biofilm cells were scraped, transferred with the saline to a centrifuge tube and centrifuged at 8,000 x *g* for 5 min at 4°C. Pelleted cells were flash frozen in liquid nitrogen and stored at -80°C until further use.

### RNA isolation

RNA was isolated by following the protocol previously described [4]. The isolated RNA was purified using Bio-Rad spin column (Cat. No. 732–6250, Bio-Rad, Hercules, CA) and treated with DNase I (Cat. No. R1013, Zymo Research, Irvine, CA) to remove any genomic DNA contamination. The integrity and quality of RNA samples were analyzed on Agilent Bioanalyzer 2100 (Agilent Technologies, Santa Clara, CA) using RNA 6000 Pico kit (Cat. No. 5067–1513, Agilent Technologies, Santa Clara, CA). Bacterial rRNAs were removed using Life technologies MICROBExpress kit (Cat. No. AM1905, Grand Island, NY) and the extent of the removal of rRNA from the samples was analyzed on Agilent Bioanalyzer 2100.

### Library construction and sequencing

To generate RNA optimal for sequencing rRNA-depleted RNA samples were fragmented to ~100 nt using NEBNext Magnesium RNA fragmentation module (Cat. No E6150S, NEB, Ipswich, MA). Strand specific cDNA libraries were prepared by treating fragmented RNA sample to phosphatase followed by T4 RNA ligase (NEB, Ipswich, MA). The 5’ and 3’ adapters were ligated and reverse transcribed (Cat. No. 18080–400, Invitrogen, Grand Island, NY) using 3’ adapter specific reverse transcriptase primer sequences. The resulting product was PCR amplified with barcoded reverse primer for 15 cycles and subjected to polyacrylamide gel extraction to purify the cDNA (50 to 200 nt) devoid of library adapters and primers. The purified cDNA library was analyzed on an Agilent Bioanalyzer 2100 using the high sensitivity DNA kit (Cat. No. 5067–4626, Agilent Technologies, Santa Clara, CA) and quantitated by qPCR as recommended by the manufacturer (Illumina). The cDNA libraries were pooled and subjected to sequencing on an Illumina HiSeq2500. For each condition, we used two biological and two technical replicates. The adapter sequences, reverse transcriptase and PCR primers are listed in Table 1. The RNA-seq data have been deposited at NCBI (SRP052780; http://www.ncbi.nlm.nih.gov/sra).

**Table 1:** Primers used in RNA-seq.

Description	Primers
**5’ Adapter**	5’ GUUCAGAGUUCUACAGUCCGACGAUCNNNN 3’
**3’ Adapter**	5’/5rApp/NNNNTGGAATTCTCGGGTGCCAAGG/3ddC 3’
**RT primer**	5’ GCCTTGGCACCCGAGAATTCCA 3’
**PCR primer (F)**	5’AATGATACGGCGACCACCGAGATCTACACGTTCAGAGTTCTACAGTCCGA 3’
**PCR primer (R) (barcode)**	5’CAAGCAGAAGACGGCATACGAGATCGTGATGTGACTGGAGTTCCTTGGCACCCGAGAATTCCA 3’

### Computational analysis

The RNA-seq analysis was performed using CLC Genomics workbench version 7.5 (Boston, MA). The Illumina 100 bp single reads were trimmed based on the quality (5 bp at the 5’ end and 25 bp at the 3’ end of the reads) and the resulting 70 bp was used for mapping using the genome of *A*. *actinomycetemcomitans* strain D7S-1 (www.kegg.jp; file NC_017846.gbk) [18, 19]. The aligned reads from each sample were visualized with CLC Genomics viewer to confirm the quality of the alignment. The gene expression values were calculated as “read per kilobase of exon unit per million mapped reads” (RPKM) [20]. The RPKM values from IDH781 and EA1002 groups were used to calculate the differential gene expression on the basis of a negative binomial distribution with R package DESeq version 1.16.0 [21]. The EDGE test was performed to get statistically significant changes and a list of genes was filtered using a P-value of <0.05 and a fold-change of >1.5 [22, 23]. The annotation and gene ontology (GO) terms were downloaded from the Uniprot website (Uniprot.org) and the information was added to the existing files in CLC Genomics for annotation test.

The overrepresented GO annotations in the differentially expressed genes were identified by comparing to the broader reference assembly using the gene set enrichment analysis (GSEA) tool in CLC Genomics workbench [24]. As implemented, GSEA considers a measure of association between the genes and phenotype of interest (e.g. test statistic for differential expression). The test implicitly calculates and uses a standard *t*-test statistic for two-group experiments as a measure of association. The P-values for the GSEA test statistics are calculated by permutation. The perturbed categories derived from the GSEA were chosen for further analysis. When necessary, the web-based Comparative GO analysis was performed [25]. This analysis has been designed for bacterial genomes using a combined graphical and statistical comparison. The KegArray analysis was used for pathway mapping (Version 1.2.3; [18]). Such combined analyses were used to obtain a comprehensive understanding of the various biological processes that are different between the two strains. Statistical parameters were generated using GSEA, CLC Genomics or Comparative GO analysis and genes were mapped using KEGG PATHWAY maps.

### Quantitative RT-PCR

Total RNA was isolated as described previously [4] and the removal of DNA was confirmed in all the samples by PCR using primers for the adhesin gene *apiA* (Table 2) before the qRT-PCR analysis. The samples were reverse transcribed using the high capacity cDNA synthesis kit containing random hexamers from Life Technologies (Grand Island, NY) following manufacturer’s instructions. To detect mRNA the cDNA was diluted 1:15 whereas to detect 16S rRNA cDNA was to 1:7000. The selected gene targets (Table 2) were amplified with Roche SYBR green master mix using the Light Cycler 480 system (Roche, Indianapolis, IN). The data were analyzed using the Light Cycler 480 software (Version 1.2.9.11), using 16S rRNA as internal control. The differential gene expression between IDH781 and EA1002 strains was calculated based on 2^-ΔΔCт^ value compared to 16S rRNA using three biological replicates each of which had two technical replicates. The results were subjected to Student’s *t*-test for statistical significance (P < 0.05).

**Table 2:** Primers used in qPCR experiment.

Gene	Forward Primer	Reverse Primer
***nagZ* (D7S_01975)**	5’ GCTTACAACGCCTATTCAAACG 3’	5’ CATTGCTGTTGAAGTGCGG 3’
***oppA* (D7S_00668)**	5’ GCCTTATCTTGCAGCGTTTATG 3’	5’ CGACTTCTGCTTCTGGGATC 3’
***ampG* (D7S_00483)**	5’ TCAAAAGCACTTCCCTCTGG 3’	5’ ACGAAACCGAAGACCCATAAG 3’
***murA* (D7S_02021)**	5’ AAACCGGGACATTCTTAGTGG 3’	5’ CGTGATGGTATCTTCGGTGAC 3’
***amiB* (D7S_02275)**	5’ AAAAGCAAGGACGAAAACACC 3’	5’ CGGGTTCAGTTTTAGCAAGG 3’
***manZ* (D7S_01286)**	5’ TTGCCTATTCCATGGTACCG 3’	5’ CAATTGCCACGCCTAATACG 3’
***mreB* (D7S_00841)**	5’ AATCGGTATCGCCTACATTCAG 3’	5’ TCGCTTCTAACACATCACGG 3’
***pgm* (D7S_01629)**	5’ CCCCGGCAGATAAACAAATTG 3’	5’ GTTCTTTGGCAAGTGAAGCG 3’
***glmS* (D7S_00991)**	5’ TGATCGGTTTAGGGATTGGTG 3’	5’ GACGGGTAATTTCGGCAATG 3’
***apiA* (D7S_00446)**	5’ CTCTACATCAGCCTTATCCGC 3’	5’ TGATGGAAATCTGGAAGGCG 3’
**16S rRNA**	5’ AAATGCGTAGAGATGTGGAGG 3’	5’ TATCTAATCCTGTTTGCTCCCC 3’

### Confocal microscopy and cell viability

For confocal microscopy, the bacterial strains were grown on 35 mm glass bottom microwell culture dishes (Cat. No. P35G-1.5-14-C, MatTek Co. Ashland, MA) as previously described [4]. For this study, biofilms were grown for 16 h, 24 h or 48 h, washed with prewarmed (37°C) fresh TSB medium and stained with Film tracer biofilm LIVE/DEAD stain (Cat. No. L7007, Life Technologies, NY). Stained biofilms were washed with fresh prewarmed (37°C) TSB medium and the images were acquired with a Nikon A1R-A1 confocal microscope (Nikon Instruments Inc., Melville, NY) using the objective lens Plan Apo VC 60x WI DIC N2. The images were processed and the percentage of LIVE/DEAD cells was calculated from seven random zones (NIS Elements software Version 4.2) expressed as a ratio of dead/total cells x 100. The results were subjected to Student’s *t*-test for significance (P < 0.05). For cell viability experiments, biofilm cells collected at 16h or 48 h were suspended in 1 ml of TSB medium and adjusted to an OD_600_ of 1.0. Cells were serially diluted and plated on TSA plates and incubated for 2 days after which the colonies were counted. The colony counts were subjected to Student’s *t*-test (Graph Pad Version 5) using means of biological triplicates.

### Electron microscopy

Biofilm cells (IDH781 and EA1002) were grown as described above and fixed overnight using a mixture of 2.5% glutaraldehyde (v/v) and 4% paraformaldehyde in 0.1 M cacodylate buffer (pH 7.4) at 4°C. The glutaraldehyde/paraformaldehyde was removed by vacuum aspiration, the bacterial cells were washed once with PBS, and then dehydrated through a graded ethanol treatment. The cells were removed from the dishes by using propylene oxide. The floating biofilm layers were washed with several changes of propylene oxide, pelleted by low-speed centrifugation, infiltrated and embedded in Epon resin. Ultrathin sections (97 nm) were prepared on an LKB III ultramicrotome, sections were picked up on a 200 mesh copper grid and stained with uranyl acetate and lead citrate. Electron micrographs from a Philips CM12 electron microscope (80 kV; Philips, Eindhoven, The Netherlands) were obtained using an AMT digital camera.

### Glycogen staining using iodine

Bacterial cells (IDH781 and EA1002) grown on TSA plates for 2 days were collected by scraping and suspended in PBS buffer. The OD_600_ was adjusted to 0.8 and 100 μl of the uniformly distributed cells was diluted to 25 ml using modified Kornberg medium (1.1% K_2_HPO_4_, 0.85% KH_2_PO_4_, 0.8% yeast extract (pH 6.8), 1% glucose and 0.4% sodium bicarbonate). Biofilms were grown in 96 well Falcon tissue culture plates (Cat. No. 353072, Corning, NY) using 100 μl of diluted cells in each well for 16 h in 10% CO_2_ incubator. After 16 h, biofilms were washed twice with prewarmed PBS buffer (37°C) and stained with 0.05 M iodine solution for 10 min. The excess iodine was removed, the biofilms were washed in running water (x3), dried and photographed. The iodine staining was performed in triplicate for each strain. Control wells had only the buffer with no inoculum.

## Results

### Transcriptome analysis

**Fig 1:**
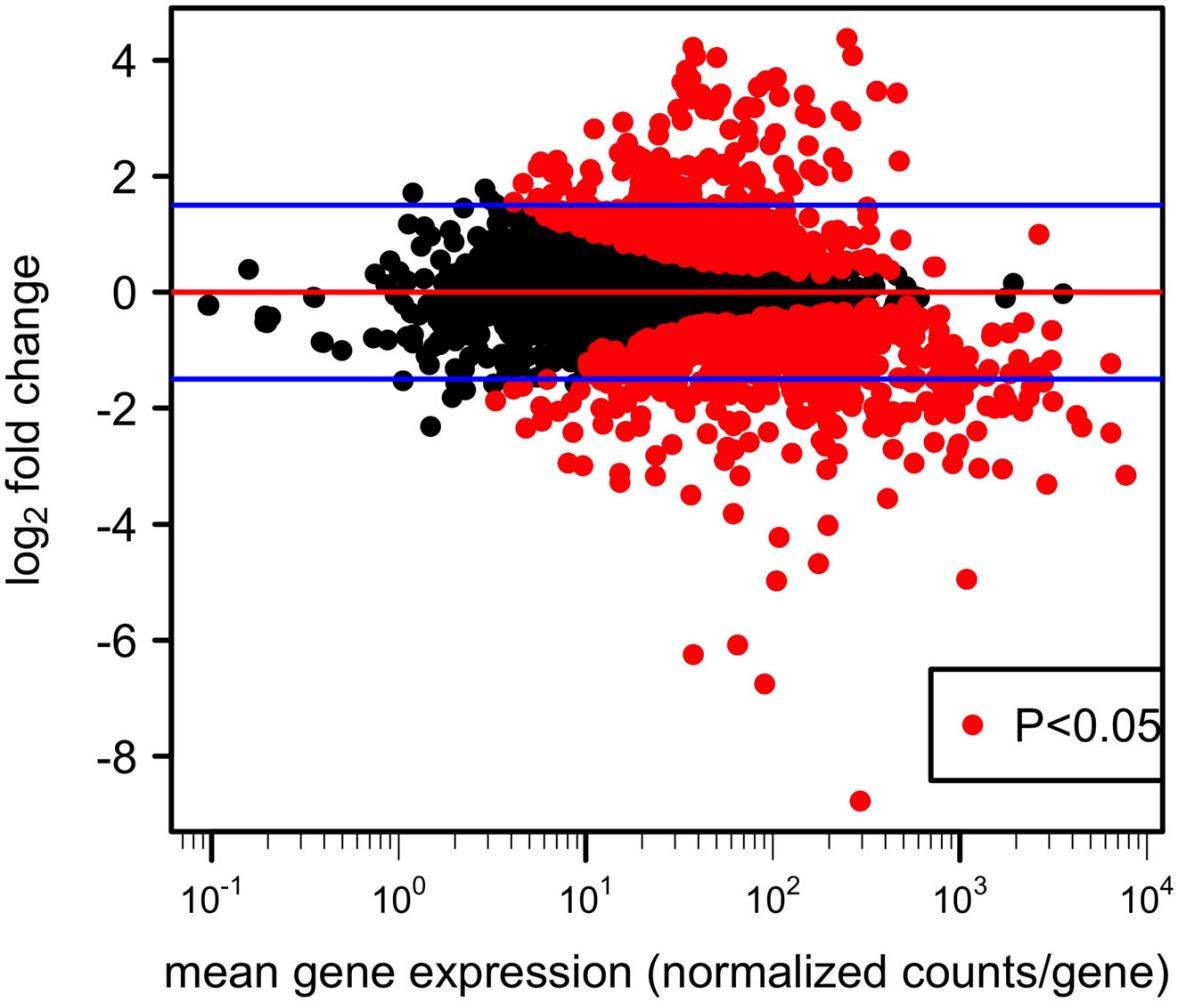
Differential gene expression plot. The fold change in the expression of each gene is plotted against mean gene expression. Red points represent genes with significant differential expression in IDH781 and EA1002 (P < 0.05). The blue bars indicate log_2_ fold change at 1.5.

To investigate the role played by PGA in *A*. *actinomycetemcomitans*, transcriptome analysis was conducted with both IDH781 and EA1002 samples taken at 16 h under *in vitro* biofilm condition. Our main goal was to use RNA-seq to obtain changes in pathways that might be impacted in the absence of *pga* genes. The *A*. *actinomycetemcomitans* genome sequence available for the strain D7S-1 was used for mapping and to identify differentially expressed genes by comparing IDH781 and EA1002 transcriptomes. Gene expression data were normalized among all replicates for differential expression analyses. A total of 1123 genes (out of 2261) were found to be differentially expressed (>1.5-fold change with a P-value less than 0.05). Of these, 579 were down-regulated and 544 genes were up-regulated in IDH781 cells compared to EA1002 (Fig 1; S1 Table). As expected, since we used a knock out strain for comparison, the *pga* operon genes were higher in IDH781 by as much as 4800-fold (*pgaC*; P = 4E-162).

**Fig 2:**
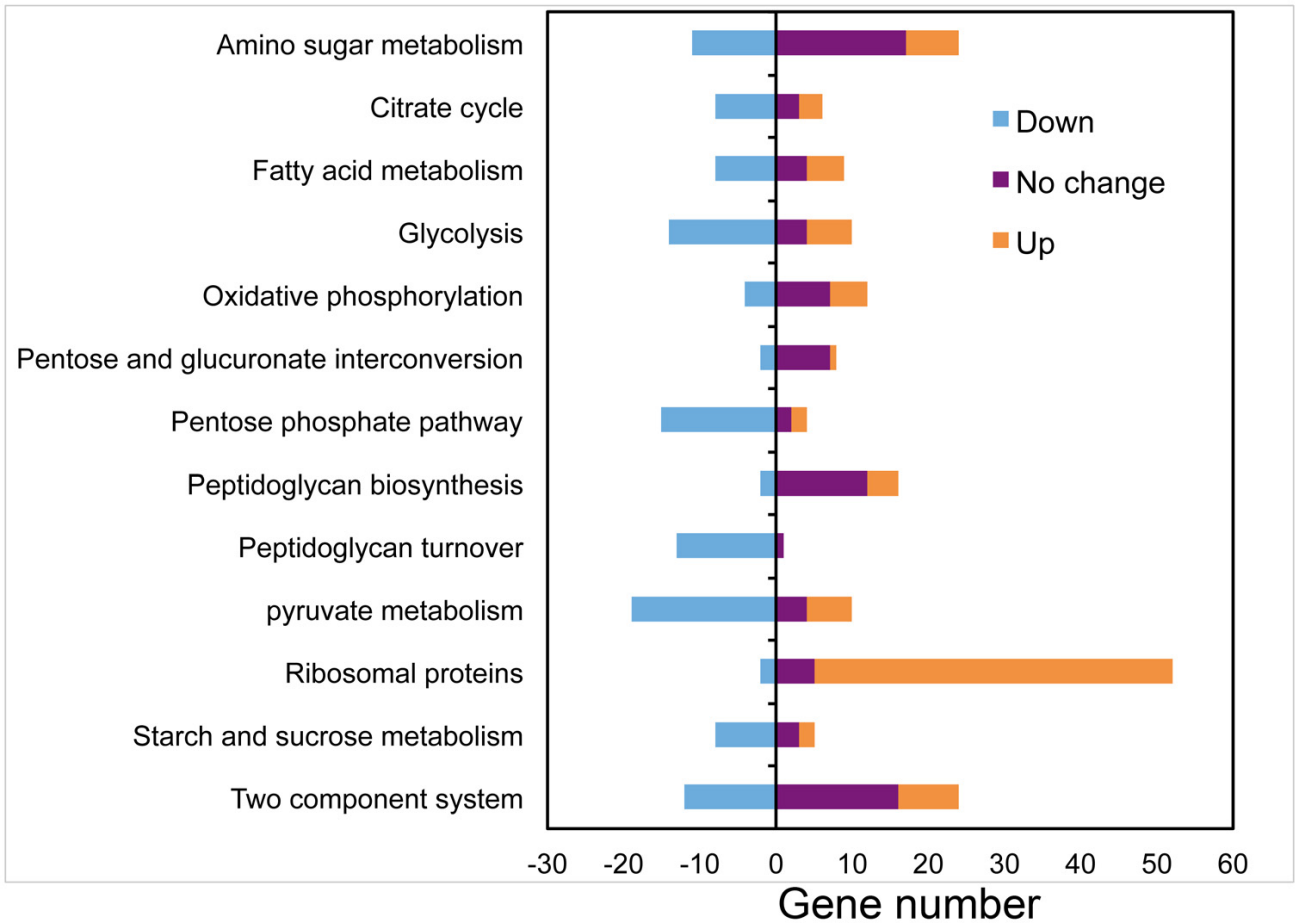
Pathway analysis plot. Number of up- and down-regulated genes in *A*. *actinomycetemcomitans* IDH781 strain. In this comparison, up-regulated genes in the the mutant strain EA1002 deficient in PGA synthesis are shown in light blue. Data are grouped according to the major role categories. Positive and negative numbers correspond to the up- and down-regulated genes, respectively. Statistical values for the various pathways are provided in S2 and S3 Tables.

To gain a better perspective of the various pathways and genes that were enriched, the software KegArray (Version 1.2.3; [18]) was used for the pathway analysis and to group the differentially expressed genes according to function (Fig 2). Furthermore, gene enrichment analysis was also computed with the CLC Genomics GSEA tool [24] to obtain statistical values associated with each pathway. When necessary, we also utilized the Comparative GO analysis for further statistical parameters associated with each pathway [25]. Using such combined analyses, we observed that genes associated with amino sugar, fatty acid, pyruvate and starch/sucrose metabolism, as well as glycolysis, pentose phosphate pathway, peptidoglycan turnover, citrate cycle, and two-component system are all up-regulated in EA1002 to a significant extent (Fig 2; light blue bars). This suggests that strain EA1002 undergoes a substantial change in metabolism during biofilm growth. Particularly noteworthy change occurred with the ribosomal protein coding genes where almost all of these were down-regulated in EA1002 compared to IDH781.

### Energy metabolism

**Fig 3:**
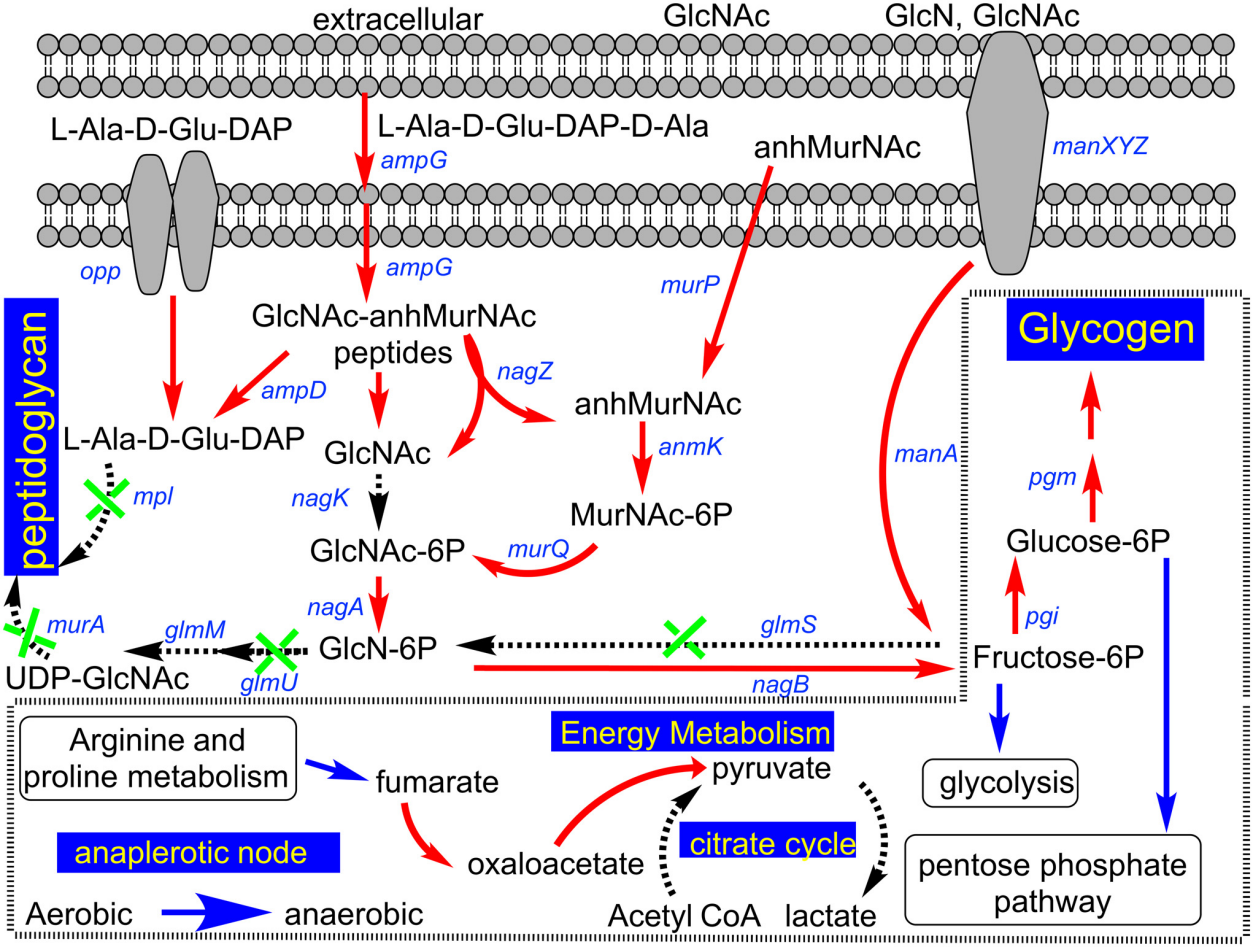
Pathway analysis. A schematic presentation of metabolic pathways affected in *A*. *actinomycetemcomitans* (IDH781 and EA1002) strains. Red arrows indicate up-regulated genes in EA1002 while dashed black arrows indicate no change in expression. Green crosses on dashed black arrows refer to down-regulated genes whereas the blue arrows indicate intermediates feeding into a pathway.

The energy producing pathways are drastically affected in the absence of *pga* genes in EA1002. Genes involved in glycolysis, the first phase of major carbohydrate catabolism, are up-regulated in EA1002 including the gene coding for the rate limiting step enzyme (6-phosphofructokinase, D7S_01972, 2.3-fold, P = 5.9E-14; Fig 3; S1 Table). Similarly, there is up regulation of pentose phosphate pathway genes including the gene coding for glucose-6-phosphate-1-dehydrogenase (D7S_00813, 1.9-fold, P = 6.7E-12; S1 Table). On the other hand, the genes for enzymes involved in the anaplerotic node [26] are all up-regulated in EA1002. In fact, fumarate generated by arginine and proline metabolism, is converted into oxaloacetate (Fig 3). This fumarate may be used to replenish oxaloacetate that was withdrawn for anabolic purposes. The genes for enzymes involved in the conversion of fumarate to oxaloacetate, malate dehydrogenase (D7S_00227; 2.5-fold, P = 5.2E-16), fumarate hydratase class II (D7S_00567; 3.4-fold, P = 3.4E-14) and fumarate reductase subunits (D7S_01501 through D7S_01504; 9–12-fold, P < E-64; only the highest P-value for the group is shown) are highly up-regulated in EA1002.

The genes for enzymes involved in the production of pyruvate from Acetyl-CoA, which is generated from various other pathways, are all down-regulated in EA1002 (D7S_00312, 1.9-fold, P = 3.5E-10 and D7S_00314, 4.1-fold, P = 4.1E-42). In EA1002, the genes for enzymes that convert pyruvate to L-/D-lactate or vice versa (L-lactate dehydrogenase, D7S_00496; 4.5-fold, P = 1.5E-44 and D-lactate dehydrogenase, D7S_00686, 1.7-fold, P = 1.7E-06) are also down-regulated. On the other hand, the gene for the enzyme phosphoenolpyruvate carboxykinase that converts oxaloacetate to phosphoenol pyruvate is down-regulated (D7S_00983, 2.0-fold, P = 4.4E-09) in EA1002. Nevertheless, pyruvate is generated from oxaloacetate by three enzymes (oxaloacetate decarboxylases D7S_00029, D7S_00030 and D7S_00031), whose genes are up-regulated in EA1002 (6 to 14-fold, P < E-20). Thus, a lack of PGA production in EA1002 is countered with a metabolic shift from aerobic (IDH781) to anaerobic mode (EA1002) for energy production (Fig 3).

### Peptidoglycan recycling and cell viability

The GSEA analysis of over represented GO terms revealed the perturbation of peptidoglycan turnover genes in EA1002. We applied knowledge-based approach (see Methods) to collect the information on genes involved in peptidoglycan turnover pathway. This approach allowed us to identify most of the genes affected in peptidoglycan turnover in the strain EA1002 (Fig 3). Several critical enzymes coded by the genes in the pathway are down-regulated in EA1002. For example, genes for enzymes involved in the conversion of fructose-6-P to UDP-GlcNAc (D7S_00991, *glmS*, 2.0 fold, P = 2E-11; D7S_01247, *glmU*, 2.0 fold, P = 1.3E-11) are down-regulated. Also, the crucial peptidoglycan precursor synthesis gene *murA* is down-regulated in EA1002 strain (D7S_02021, 1.5-fold, P = 1.8E-05). Further down the pathway, gene for another enzyme UDP-N-acetylmuramate-alanine ligase is also down-regulated (D7S_01482, 1.5 fold, P = 6.7E-06). In addition, the gene for bactoprenol-linked glucose translocase is also down-regulated (D7S_02278, 1.8 fold, P = 0.01). Thus, peptidoglycan synthesis and turnover is likely attenuated in EA1002.

**Fig 4:**
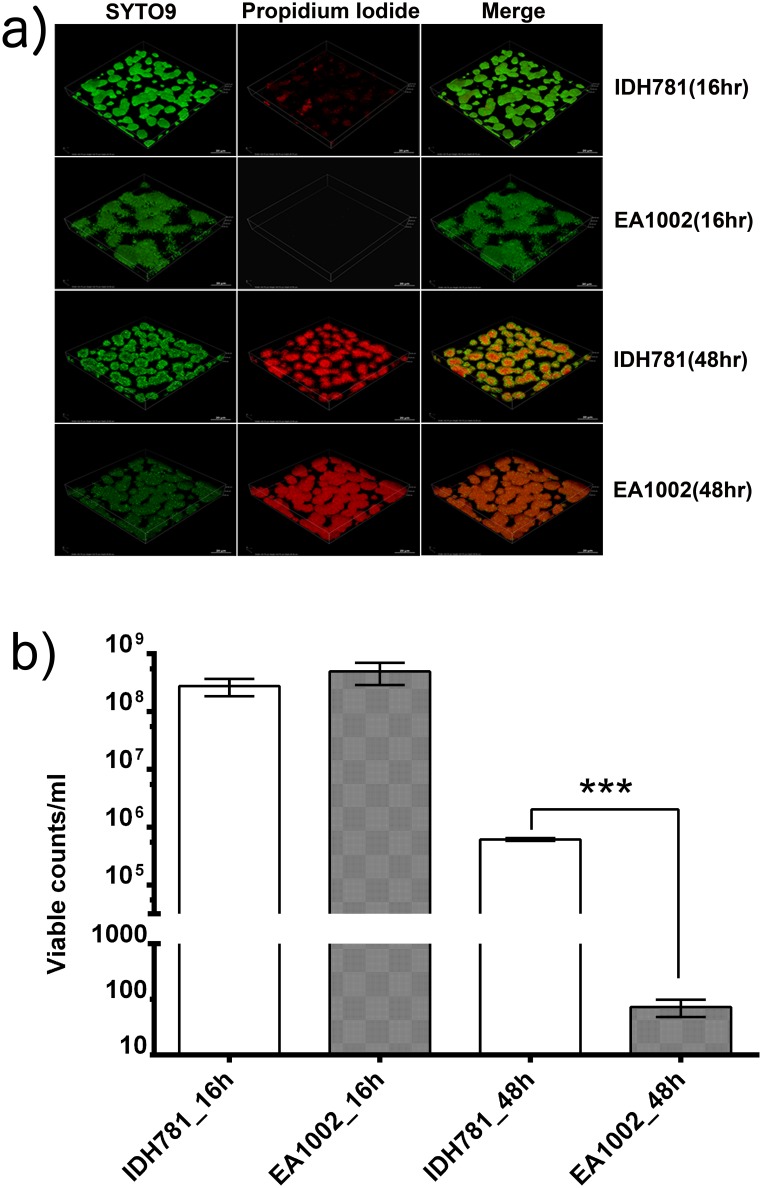
Confocal imaging analysis. a) Live/Dead staining of cells at 16 h and 48 h. Cell death (propidium iodide stain, red channel) is substantially higher at 48 h than at 16 h. At 48 h, however, there is significantly more death in EA1002 cells and the cell death is apparent throughout the biofilm colonies. In contrast, in IDH781 cells, there are more live cells at the periphery than in the middle. Cell death for the strains were comparable and similar to cell death observed at 16 h (S1 Fig). b) Cell viability for IDH781 and EA1002 strains at different time intervals of growth.

To evaluate whether the apparent attenuated peptidoglycan turnover affects the cell viability, both IDH781 and EA1002 cells grown for 16h and 48 h were analyzed. Syto 9/propidium iodide (Live/Dead) staining was used to compare the amount of cell death using confocal microscopy. The Live/Dead staining showed that while the cell death at 16 h was comparable between the two strains (IDH781 5.7±0.4% vs. EA1002 0.5±0.1%), there was drastic difference at 48 h (IDH781 71±2% vs. EA1002 99±1%; Fig 4). The cell death at 48 h for EA1002 was significantly higher (Student’s *t*-test, P < 0.001 Fig 4) and was distributed throughout the biofilm colonies. In contrast, in IDH781, cell death was observed in the interior of the biofilm whereas the exterior was enriched with live cells. To confirm the excessive cell death occurring in the EA1002 strain at 48 h and to estimate the amount of viable cells present in comparison to IDH781 strain, we grew the two strains ensuring that equal number of cells were used for growth. After 16 and 48 h, IDH781 and EA1002 cells were enumerated by colony counting (Fig 4b). Clearly, there was no significant difference between the numbers of viable cells at 16 h (P > 0.17) where as there were significantly more viable IDH781 cells than EA1002 at 48 h (P < 0.0001). To test whether or not the cells were at an intermediate time point in their growth, Live/Dead staining for cells grown for 24 h were performed (S1 Fig). The cell death for both IDH781 and EA1002 cells were comparable (IDH781 1.2±0.2% vs. EA1002 0.2±0.02%; P <0.0001; Figure in S1 Fig).

### Glycogen accumulation in EA1002

The transcriptome analysis suggested that the genes involved in the accumulation of glycogen were up-regulated in EA1002. In this regard, anhydromuropeptides released during growth by lytic transglycosylase (D7S_02200; moderately up-regulated, 1.4-fold, P = 0.0006) could be imported by AmpG whose gene is up-regulated (Fig 3; D7S_00483, 2.4 fold, P = 1.4E-05). In the cytoplasm, this could increase the availability of GlcNAc through the action of NagZ (*nagZ*, D7S_01975, up-regulated by 2.3-fold, P = 1.9E-09). However, subsequent conversion to GlcNAc-6P is limiting because of NagK (*nagK*, D7S_00404, no significant change). Nevertheless, GlcNAc-6P is likely to be accumulated through another route involving enzymes AnmK (*anmk*, D7S_01246, 2.7-fold, P = 6.1E-08) and MurQ (*murQ*, D7S_01245, 5.6-fold, P = 5.8E-08). The GlcNAc-6P is subsequently converted to fructose-6-phosphate because GlmS is down-regulated (*glmS*, D7S_00991, 2.0-fold, P = 1.9E-11) compared to NagB (*nagB*, D7S_00401, no change). In essence, the accumulation of fructose-6-phosphate results in the increase in glycolysis or pentose phosphate pathway or the accumulation of glycogen.

**Fig 5:**
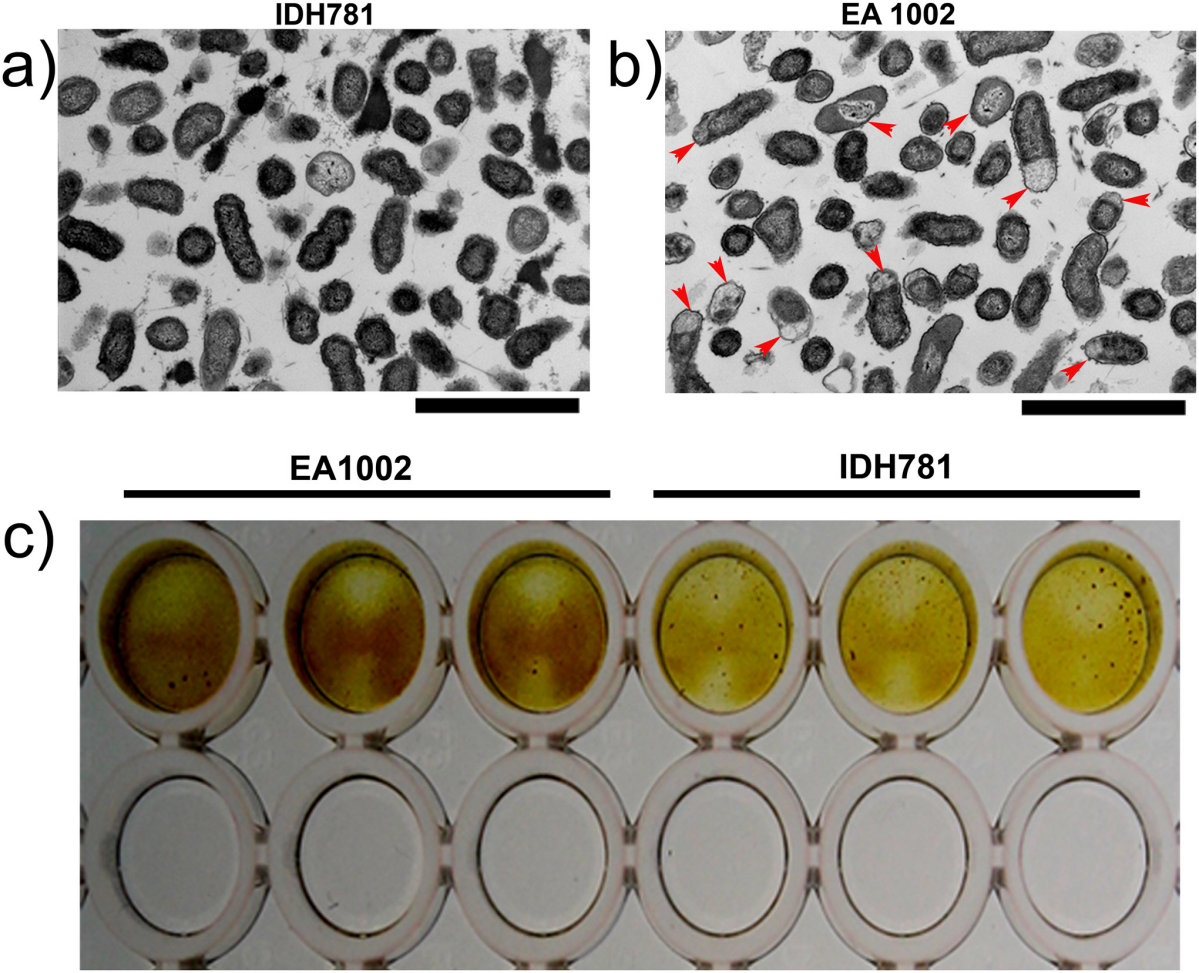
Transmission electron microscopy. a) IDH781 cells; b) EA1002 cells showing lucent regions in the periphery corresponding to accumulation of glycogen (red arrows). Magnification: x5000; Scale: 2 micron; c) Accumulation of glycogen in EA1002 shown using iodine staining. The EA1002 and IDH781 cells are shown in triplicate. The bottom row of wells is control wells without any bacterial inoculum and stained with iodine.

To test whether or not there are any physiological changes relevant to possible accumulation of glycogen, IDH781 and EA1002 cells grown as biofilms for 16 h were analyzed using electron microscopy. Transmission electron micrographs of the two strains showed that in EA1002 cells there is clear compartmentalization in the cells (Fig 5b). These regions of the cells indicated by red arrows stain lighter than the rest of the cell suggesting that there is accumulation of glycogen in EA1002 [27]. To further confirm that the EA1002 cells accumulate glycogen, more than IDH781 cells, we grew the two strains under biofilm conditions in 96 well tissue culture plates and after 16 h of growth, the wells were stained using iodine (Fig 5c). The EA1002 biofilm stains darker compared to IDH781 cells suggestive of the presence of excess glycogen. Control wells that were coated with medium alone did not stain with iodine.

### Validation of transcriptome analysis data—qRT-PCR

The mRNA expression level of select genes involved in the peptidoglycan recycling modulated by the lack of the *pga* operon was determined. The rationale for selecting these genes was based on their product enzymes that are either rate limiting or important in a given pathway. The enzymes in various pathways selected for this analysis were: N-acetylglucosamine recycling, μ-hexosaminidase, NagZ; oligopeptide recycling, periplasmic ABC transporter, OppA; murein recycling, GlcNAc-anhMurNAc permease, AmpG; peptidoglycan precursor synthesis, UDP-N-acetylglucosamine-1-carboxyvinyl transferase, MurA; peptidoglycan recycling or autolysis, N-acetylmuramoyl-L-alanine amidase, AmiB; aminosugar transport, PTS system, ManZ; cell size determination proteins, rod-shape determining protein MreB; D-fructose-6-phosphate amidotransferase, GlmS; and phosphoglucomutase/phosphomannomutase *pgm*.

**Fig 6:**
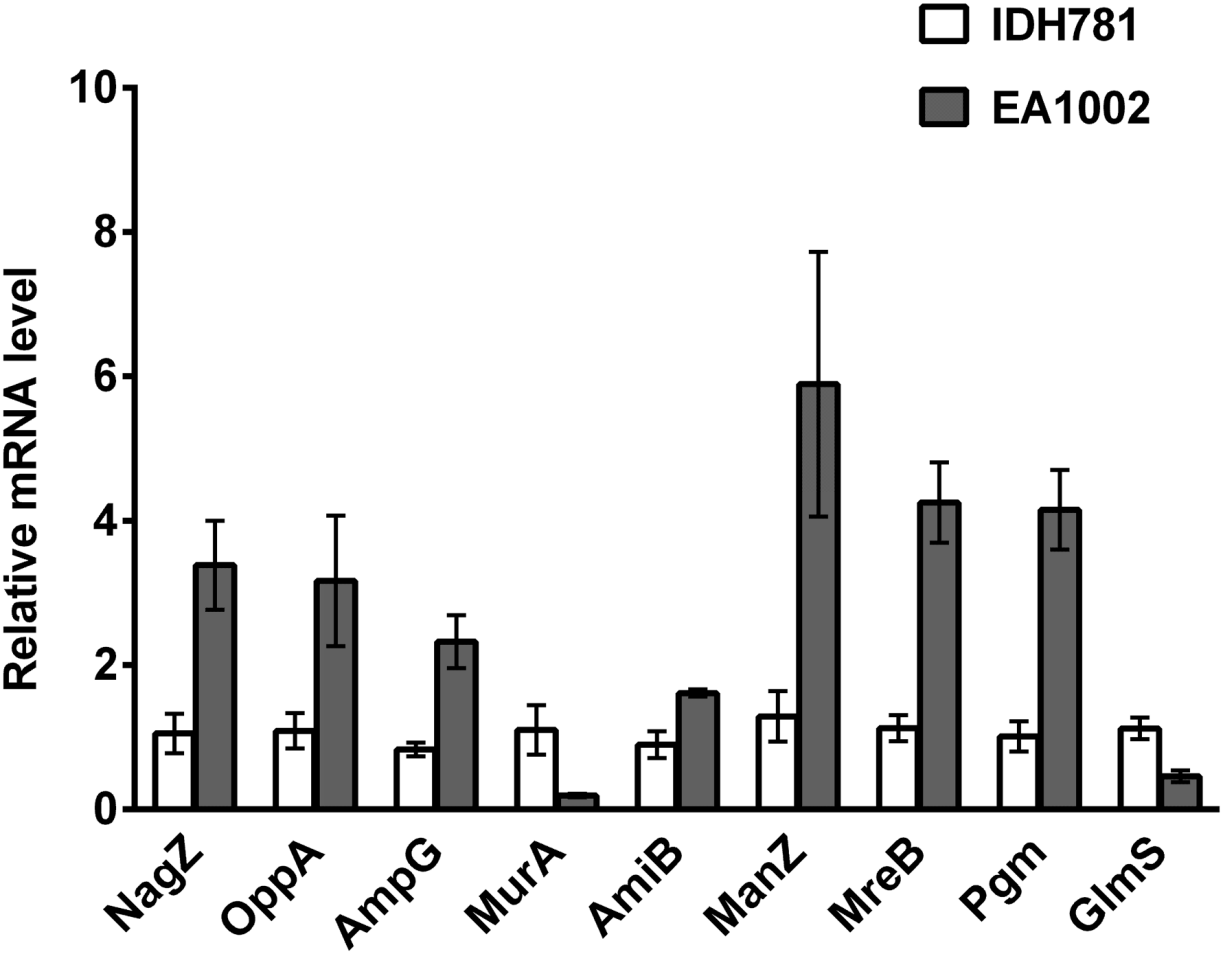
Expression of select genes in the *A*. *actinomycetemcomitans* strains. The mRNA was isolated, and relative levels of the genes critical in peptidoglycan synthesis (*murA*, *glmS*), glycogen synthesis (*pgm*) and other genes shown were quantified by qPCR as described in the text. Results are means ± standard deviations for triplicate cultures normalized to 16S rRNA. The fold changes in the expression levels of all represented genes are significantly different between the strains as measured by Student’s *t-test*.

For qRT-PCR analysis, mRNA was isolated, transcribed into cDNA, and subjected to qRT-PCR. The results demonstrate that the critical genes for the peptidoglycan synthesis are down-regulated in EA1002 (Fig 6; see Peptidoglycan recycling and cell viability section above). Some genes involved in glycolysis and glycogen synthesis (*pgm* for example) are significantly up-regulated in EA1002 (lack of production of PGA) except for *murA* (Fig 6). The fold changes in the mRNA levels between IDH781 and the EA1002 strains in other genes such as *aae*, *apiA*, *flp-1*, which are involved in colonization, have previously shown to be affected as well [4]. Interestingly, the immune evasion genes, *ltxA* and *cdtB* were not significantly different [4]. Significance in the fold changes was analyzed using Student’s *t*-test and the P-values ranged from 0.0008 (*mreB*) to 0.018 (*oppA*). In the RNA-seq analysis, the colonization genes were significantly down-regulated (*flp-1*, D7S_01457, 2.5-fold, P = 8.7E-18; *apiA*, D7S_00446, 30.6-fold, P = 3E-154; and *aae*, D7S_2013, 1.5-fold, 5.4E-05) while the toxins were not (*ltxA*, D7S-00604, 1.2-fold, P = 0.13 and *cdtB*, D7S_02295, 1.3-fold and P = 0.012). Thus, the phenotypic/physiologic changes we observed in the EA1002 are likely to be reflective of the changes that occur in the pathways as outlined in Fig 3.

## Discussion

The aim of the present study was to assess the role of PGA (*pga* genes) in the pathophysiology of the periodontal pathogen *A*. *actinomycetemcomitans*. In an earlier study we demonstrated that the absence of PGA rendered the bacterium ineffective in colonization, decreased its ability to cause bone resorption and caused a down-regulation of select virulence genes analyzed by qRT-PCR [4]. To better understand the state of the cells grown in biofilm-inducing conditions, we grew IDH781 and EA1002 cells under *in vitro* conditions for an extended period (48 h) and tested for the viability of the cells using Live/Dead stain (Fig 4a). The confocal images taken at 16 h, 24 h and 48 h showed surprising results for EA1002. At 16 h and 24 h, EA1002 cells showed minimal cell death whereas at 48 h there was massive cell death (Fig 4a and S1 Fig). In sharp contrast, IDH781 cells showed significantly less cell death at 48 h. The cell viability decrease for EA1002 observed in confocal imaging was confirmed by plating (Fig 4b).

Since PGA production is completely absent in EA1002, we surmised that such a low viability might be linked to the lack of PGA. In this regard, in other bacteria such as *Pseudomonas aeruginosa*, absence of exopolysaccharide alginate has been shown to produce a decreased proportion of viable cells [28]. Another possibility for the increased cell death could be the attenuated peptidoglycan synthesis. Inhibition of *murA* in *E*. *coli* has previously been reported to increase hydrostatic pressure inside the cell, likely increasing the chance for cell death [29]. This enhanced cell death in EA1002 prompted us to explore the changes in the differential expression of genes involved in various metabolic pathways due to the lack of the *pga* operon in *A*. *actinomycetemcomitans*. To this end, we performed a high-resolution transcriptome analysis of the two strains IDH781 and EA1002 by RNA-seq. Previously, we had shown that *A*. *actinomycetemcomitans* cells are in the exponential phase at 16 h under the conditions used in this study [30]. Therefore, we choose 16 h for our analysis. Also, since most of the EA1002 cells are dead at 48 h (Fig 5) this time point was not relevant for RNA-seq.

A number of genes were observed to be differentially expressed between IDH781 and the EA1002 cells (Fig 1). Interestingly, a significant outcome of the study is that the lack of the *pga* operon affects several metabolic pathways, especially the energy metabolism and the peptidoglycan recycling (Fig 2). We note that while growth conditions for both IDH781 and the EA1002 were the same, metabolic processes active in EA1002 resemble those of anaerobic growth rather than aerobic growth (Fig 3). Interestingly, in a recent study using transcriptome analysis, it was shown that *A*. *actinomycetemcomitans* adopts to anaerobic growth in the murine abscess model (*in vivo*) wherein the energy production and conversion is significantly modified [17]. The enrichment of pathways involving formate dehydrogenase and fumarate reductase operons were observed *in vivo* using RNA-seq [17]. In our study, the genes for these enzymes are highly up-regulated in the EA1002 (formate dehydrogenase H, 4.0 fold and P = 6.3E-38; fumarate reductase genes, ranging from 9.0–13.5 fold and P-value ranging from 1.8E-64 to 4.6E-111) suggesting that EA1002 might be experiencing low oxygen levels more than experienced by IDH781 under similar growth conditions. Such switching between aerobic and anaerobic growth is not uncommon for *A*. *actinomycetemcomitans* and might depend upon the presence of other oral bacteria [31]. What is surprising is that the EA1002 cells die after 48 h during biofilm growth indicating the shift is dramatic enough that the EA1002 cells could not sustain growth compared to IDH781 cells under similar conditions. As a corollary to these observations, one could interpolate the importance of PGA for the survival of IDH781 (as is discussed below).

The carbon storage regulator, CsrA, encoded by *csrA*, is a small RNA-binding protein that can alter translation and stability of target mRNA sequences, in particular the glycogen biosynthesis gene *glgC* in *E*. *coli* [32]. The gene *csrA* is highly down-regulated in EA1002 (D7S_01018, 3.3 fold, P = 1.3E-24) while the gene for glycogen synthase (D7S_00077, 6.0-fold, P = 4.9E-63) is highly up-regulated. This finding suggests that repression of glycogen synthesis will be minimal in EA1002 [33]. Clearly, further studies, beyond the scope of this study, are warranted to understand the relationship between *csrA* and *pga* in *A*. *actinomycetemcomitans*. Nevertheless, a possible relationship between *csrA*, the *pga* operon and PGA in *A*. *actinomycetemcomitans* emerges through parallel studies performed in *E*. *coli pga* mutants, which showed that *csrA* can regulate up to 400 genes [33, 34]. These studies showed that CsrA affects *pgaABCD* genes post-transcriptionally and PGA synthesis is necessary for CsrA and other Csr signaling components to affect biofilm formation [33]. Interestingly, while both glycogen synthesis and turnover is repressed by CsrA, the biofilm formation is not regulated by *csrA* in a *pga* mutant (Δ*pgaC*) in *E*. *coli*. Since both *E*. *coli* and *A*. *actinomycetemcomitans* produce nearly identical PGA [1, 5, 35, 36], a similar mechanism might be occurring in *A*. *actinomycetemcomitans*, whereby the absence of the *pga* operon may have some impact in *csrA* down regulation in EA1002, which in turn has an effect on glycogen synthesis (Fig 5). Supporting this idea is the electron microscopy result that showed obvious compartmentalization of glycogen and clear physiologic changes in the EA1002 cells. Additional supporting evidence for the presence of higher amounts of glycogen in EA1002 cells compared to IDH781 was obtained using iodine staining of the two strains grown in the biofilm mode (Fig 5c). The iodine staining also suggested that the EA1002 had enhanced staining compared to IDH781. Thus, the physiology of EA1002 cells (Fig 5b) is likely to be linked to the down regulation of *csrA*.

Some other interesting comparisons between IDH781 and EA1002 in the differentially expressed genes lie in those related to stress. Because of the high number of genes affected by the absence of *pga*, it is likely that there is an indirect effect involving oxidative stress. For example, the gene for GAPDH is down-regulated in EA1002 alluding to this possibility (D7S_01774, 1.6 fold, P = 2.0E-6). The enzyme GAPDH is inactivated during oxidative stress and the stressed cells shift the metabolic flux from glycolysis to pentose phosphate pathway. In EA1002, there is up regulation of gene encoding for universal stress protein UspA, also involved in oxidative stress (D7S_01016, *uspA*, 2.0-fold, P = 2.7E-08).

There are significant changes in the genes involved in the citrate cycle as well. Our RNA-seq analysis suggested that intermediates in the citrate cycle might be shunted to other pathways where they might be needed. For example, since pyruvate is not produced in the citrate cycle, it is likely to be produced by other means from fumarate (Fig 3) that in turn is generated from Arginine/Proline metabolism, as genes for several enzymes in these pathways are highly up-regulated in EA1002 (S1 Table). The enzymes involved in the conversion of fumarate to oxaloacetate to pyruvate are likely to play a role in EA1002 maintaining the energy production.

We also observed that the genes involved in ribosome integrity are highly differentially expressed. Almost all the genes that produce ribosomal proteins are down-regulated in EA1002 (Fig 2). These genes encode various proteins that are involved in protein synthesis within the ribosome. The ribosomal proteins are essential for the assembly and optimal functioning of the ribosome itself. It is unclear how the down regulation of the ribosomal genes affects the EA1002 strain. We have previously shown that the growth rate of EA1002 strain is not different from that of IDH781. However, the decrease in cell viability of EA1002 cells during biofilm growth condition suggests that at later stages of growth (between 16 h and 48 h) there could be a distinct impact of the down-regulated genes in the survival of EA1002.

Overall, our study has shown that the absence of *pga* genes affect *A*. *actinomycetemcomitans* at several different fronts. Understanding how these modes of growth affect infection or how the infection affects the gene expression would increase our understanding of how *A*. *actinomycetemcomitans* causes disease. Some preliminary insight can be obtained by our previous study showing that in spite of unchanged toxin levels (*ltxA*, D7S_00604, 1.2 fold, P = 0.13 or *cdtB*, D7S_02295, 1.3 fold, P = 0.012), strains lacking PGA might be ineffective to cause bone resorption [4]. Ultimately, understanding the physiological impact of PGA in the disease pathogenesis may provide an opportunity to hinder infection and allow us to devise better therapeutic approaches based on inhibition of synthesis of PGA. Studies in this context are underway in our laboratory. If successful, such intervention strategies could be applicable to other medically relevant bacteria such as *S*. *epidermidis* and *S*. *aureus* that also produce exopolysaccharide structurally very similar to PGA [9].

## Conclusions

The main conclusion that emerges from this study is that absence of PGA has a significant effect in the way *A*. *actinomycetemcomitans* copes with growth and pathophysiology. Even while conditions are conducive for aerobic growth, there is a shift to move to anaerobic pathways for growth in the absence of PGA. Interestingly, cell viability decreases at 48 h. In addition to the important role of PGA in the *A*. *actinomycetemcomitans*-induced bone resorption by modulating expression of genes involved in the attachment stage during biofilm growth, *pga* locus appears to affect the peptidoglycan recycling as well as to induce a metabolic shift to increase glycogen storage. While previous studies highlighted the role of PGA in biofilm formation as well in immune evasion, our study demonstrates that PGA modulates the expression levels of not only virulence genes but also other genes for growth and viability. Inhibition of PGA synthesis or methods devised to repress *pga* locus might be therapeutically relevant in disease prevention.

## Supporting Information

S1 Fig Confocal imaging analysis. Live/Dead staining of IDH781 and EA1002 cells at 24 h. Cell death (propidium iodide stain, red channel) is comparable to each other and at 16h.
(TIF)

S1 Table Differentially expressed genes in EA1002 vs. IDH781. (DOC)

S2 Table Overrepresented GO terms; Hypergeometric test P-values (<0.05). EA1002.
(DOC)

S3 Table Overrepresented GO terms; Hypergeometric test P-values (<0.05). IDH781.
(DOC)
