# Supplementary figures and images for: Transcriptome Profiling of Wild-Type and pga-Knockout Mutant Strains Reveal the Role of Exopolysaccharide in Aggregatibacter actinomycetemcomitans
Source: PLoS One. 2015 Jul 29;10(7):e0134285. doi: 10.1371/journal.pone.0134285 (PMC4519337; doi:10.1371/journal.pone.0134285)

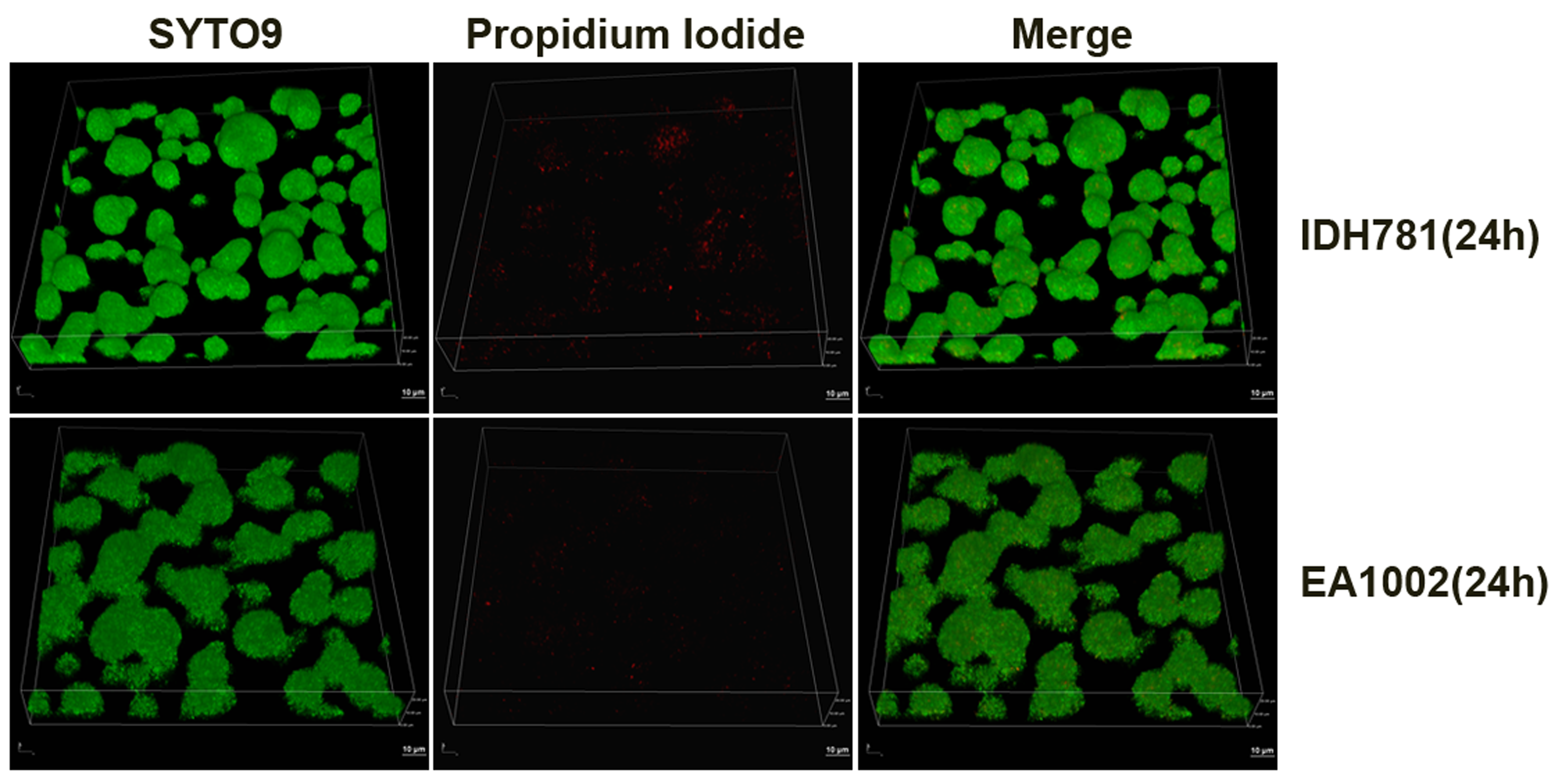

Supplement: S1 Fig — Live/Dead staining of IDH781 and EA1002 cells at 24 h. Cell death (propidium iodide stain, red channel) is comparable to each other and at 16h. (TIF) [file pone.0134285.s001.tif]
