# Supplementary material for: Transcriptome Profiling of Wild-Type and pga-Knockout Mutant Strains Reveal the Role of Exopolysaccharide in Aggregatibacter actinomycetemcomitans
Source: PLoS One. 2015 Jul 29;10(7):e0134285. doi: 10.1371/journal.pone.0134285 (PMC4519337; doi:10.1371/journal.pone.0134285)
Supplement: S1 Table — (DOC) [file pone.0134285.s002.doc]

**S1 Table. Differentially expressed genes in EA1002 vs. IDH781**

| **Feature ID** | **EDGE test: P-value** | **EDGE test:Fold change** | **Annotations** |
| --- | --- | --- | --- |
| D7S_01763 | 4.0214E-162 | 4794.50 | biofilm PGA synthesis N-glycosylltransferase |
| D7S_01761 | 4.82381E-73 | 618.35 | biofilm PGA synthesis lipoprotein PgaB |
| D7S_01857 | 3.1754E-130 | 289.41 | ComE operon protein 1 |
| D7S_01764 | 1.7724E-119 | 119.35 | WD40 repeat containing protein |
| D7S_02183 | 6.1897E-111 | 35.16 | formate dehydrogenase subunit alpha |
| D7S_00446 | 2.9872E-154 | 30.63 | outer membrane protein 100 |
| D7S_02182 | 2.2965E-122 | 26.12 | formate dehydrogenase subunit alpha |
| D7S_00503 | 0.000489494 | 23.93 | putative transporter |
| D7S_02181 | 1.5231E-113 | 19.87 | formate dehydrogenase accessory protein |
| D7S_00499 | 0.007659964 | 19.16 | hypothetical protein |
| D7S_00281 | 3.30505E-67 | 16.87 | tRNA/rRNA methyltransferase |
| D7S_01466 | 8.27368E-12 | 16.28 | phosphoheptose isomerase |
| D7S_02180 | 2.046E-98 | 14.47 | formate dehydrogenase subunit gamma |
| D7S_02179 | 2.93615E-22 | 13.52 | hypothetical protein |
| D7S_01760 | 5.81975E-61 | 12.28 | biofilm PGA synthesis protein PgaA |
| D7S_00618 | 1.80239E-20 | 11.10 | hypothetical protein |
| D7S_00619 | 1.08628E-11 | 10.30 | type II secretory pathway, pseudopilin |
| D7S_01819 | 4.1021E-128 | 9.73 | 50S ribosomal protein L22 |
| D7S_01765 | 1.07385E-29 | 9.65 | hypothetical protein |
| D7S_00617 | 3.54493E-13 | 9.49 | hypothetical protein |
| D7S_01778 | 9.23602E-82 | 8.93 | ATP-dependent DNA helicase RecG |
| D7S_02433 | 5.89274E-46 | 8.85 | DoxX family protein |
| D7S_00265 | 5.49755E-05 | 8.26 | hypothetical protein |
| D7S_01077 | 1.07547E-78 | 8.25 | 50S ribosomal protein L31 |
| D7S_02315 | 7.57804E-66 | 8.13 | PTS system glucose-specific transporter |
| D7S_01082 | 1.08933E-77 | 8.05 | 50S ribosomal protein L33 |
| D7S_01796 | 1.84323E-26 | 7.77 | hypothetical protein |
| D7S_01812 | 1.37303E-76 | 7.63 | putative HTH-type transcriptional regulator |
| D7S_00972 | 3.95985E-87 | 7.60 | hypothetical protein |
| D7S_01597 | 1.47846E-53 | 7.60 | YifE like protein |
| D7S_01101 | 4.87383E-10 | 7.35 | protein transport protein HofQ |
| D7S_01752 | 0.00161378 | 6.95 | hypothetical protein |
| D7S_00864 | 1.39418E-52 | 6.82 | Plasmid maintenance system killer |
| D7S_00951 | 8.91462E-57 | 6.80 | hypothetical protein |
| D7S_00300 | 7.03464E-05 | 6.74 | hypothetical protein |
| D7S_00083 | 2.18144E-33 | 6.51 | hypothetical protein |
| D7S_02075 | 4.58273E-52 | 6.48 | PemI-like protein |
| D7S_00685 | 9.92944E-49 | 6.48 | thioredoxin |
| D7S_00359 | 8.28564E-62 | 6.48 | hypothetical protein |
| D7S_00022 | 1.00524E-24 | 6.42 | ribonucleoside-diphosphate reductase subunit |
| D7S_01795 | 1.18863E-06 | 6.39 | hypothetical protein |
| D7S_02419 | 9.2371E-52 | 6.23 | hypothetical protein |
| D7S_00282 | 0.000332906 | 6.11 | hypothetical protein |
| D7S_01273 | 1.89449E-44 | 6.05 | hypothetical protein |
| D7S_02261 | 2.19188E-72 | 5.96 | peptide transport system ATP-binding protein |
| D7S_02203 | 5.74582E-06 | 5.94 | dTDP-glucose 4,6-dehydratase |
| D7S_00065 | 8.23783E-39 | 5.93 | nonheme iron-containing ferritin |
| D7S_00417 | 1.34757E-15 | 5.85 | TPR repeat protein |
| D7S_00454 | 1.20225E-42 | 5.82 | Ndh protein |
| D7S_01084 | 1.31335E-06 | 5.81 | DNA repair protein RadC |
| D7S_02193 | 2.97527E-35 | 5.57 | prevent-host-death protein |
| D7S_00266 | 0.018856871 | 5.37 | hypothetical protein |
| D7S_00990 | 1.54785E-31 | 5.37 | glucitol operon repressor |
| D7S_00779 | 8.94295E-42 | 5.26 | 30S ribosomal protein S4 |
| D7S_00474 | 3.20709E-48 | 5.14 | hypothetical protein |
| D7S_00272 | 9.47162E-06 | 5.14 | hypothetical protein |
| D7S_00419 | 4.84179E-14 | 5.14 | hypothetical protein |
| D7S_01019 | 3.32546E-41 | 5.00 | phosphomannomutase |
| D7S_01017 | 7.00864E-42 | 4.95 | alanyl-tRNA synthetase |
| D7S_02006 | 6.34281E-37 | 4.95 | nicotinamide phosphoribosyltransferase |
| D7S_00789 | 1.70919E-41 | 4.90 | 30S ribosomal protein S14 |
| D7S_00355 | 1.24234E-36 | 4.88 | hypothetical protein |
| D7S_01831 | 1.7567E-48 | 4.87 | 30S ribosomal protein S21 |
| D7S_02447 | 0.000785584 | 4.82 | putative tail fiber assembly protein |
| D7S_01996 | 5.05579E-43 | 4.77 | trigger factor |
| D7S_01790 | 2.46468E-36 | 4.72 | hypothetical protein |
| D7S_02420 | 1.94354E-29 | 4.70 | hypothetical protein |
| D7S_01890 | 1.77957E-15 | 4.61 | type IV pilus biogenesis/stability protein PilW |
| D7S_01563 | 6.6079E-33 | 4.53 | Chb protein |
| D7S_00496 | 1.48496E-44 | 4.50 | L-lactate dehydrogenase LctD |
| D7S_00074 | 2.85164E-38 | 4.42 | lipoprotein HlpB |
| D7S_02024 | 1.00442E-40 | 4.40 | ABC transporter |
| D7S_01312 | 2.4262E-05 | 4.39 | Rhs element Vgr protein |
| D7S_00075 | 4.49605E-37 | 4.37 | 50_S_ribosomal protein L25 |
| D7S_00699 | 0.005437513 | 4.34 | hypothetical protein |
| D7S_00343 | 1.64224E-37 | 4.29 | pyridine nucleotide transhydrogenase |
| D7S_02028 | 0.001188224 | 4.29 | hypothetical protein |
| D7S_00836 | 3.23426E-30 | 4.27 | 16S rRNA processing protein RimM |
| D7S_00769 | 0.003947232 | 4.27 | hypothetical protein |
| D7S_00616 | 0.000697312 | 4.27 | Putative type II secretory pathway, pseudopilin |
| D7S_00845 | 2.74648E-09 | 4.26 | glycerol-3-phosphate transporter |
| D7S_01997 | 5.45691E-21 | 4.25 | hypothetical protein |
| D7S_01791 | 2.07291E-41 | 4.25 | periplasmic/secreted protein |
| D7S_02224 | 2.45915E-23 | 4.24 | addiction module killer protein |
| D7S_02023 | 4.35052E-38 | 4.24 | anti-sigma factor B antagonist |
| D7S_00440 | 4.61038E-41 | 4.19 | outer membrane protein A |
| D7S_01822 | 4.98173E-26 | 4.18 | 50S ribosomal protein L29 |
| D7S_02025 | 2.06902E-40 | 4.15 | ABC transporter substrate-binding protein |
| D7S_02370 | 2.06997E-10 | 4.13 | ISPsy24, transposase orfB |
| D7S_00314 | 4.13035E-42 | 4.09 | pyruvate dehydrogenase subunit E1 |
| D7S_00554 | 4.07541E-32 | 4.08 | DNA protection during starvation protein |
| D7S_01789 | 8.08586E-42 | 4.08 | large conductance mechanosensitive channel |
| D7S_00130 | 0.030939124 | 4.05 | hypothetical protein |
| D7S_00784 | 1.81373E-43 | 4.03 | 50S ribosomal protein L15 |
| D7S_00802 | 2.46981E-06 | 3.94 | FHA domain-containing protein |
| D7S_00144 | 9.41849E-40 | 3.92 | H-NS histone family |
| D7S_00274 | 0.005177297 | 3.90 | tRNA/rRNA methyltransferase |
| D7S_00837 | 8.45172E-39 | 3.90 | tRNA guanine-N1--methyltransferase |
| D7S_00697 | 1.17461E-08 | 3.90 | ISPsy24, transposase orfB |
| D7S_00172 | 3.45824E-41 | 3.87 | 30S ribosomal protein S1 |
| D7S_00451 | 1.00907E-29 | 3.86 | S49 family peptidase |
| D7S_00848 | 4.8213E-27 | 3.84 | 50S ribosomal protein L9 |
| D7S_01182 | 6.08163E-34 | 3.83 | 50S ribosomal protein L32 |
| D7S_00792 | 1.0996E-33 | 3.80 | 50S ribosomal protein L14 |
| D7S_01322 | 5.61037E-11 | 3.79 | periplasmic nitrate reductase, large subunit |
| D7S_01818 | 2.74356E-35 | 3.74 | 30S ribosomal protein S19 |
| D7S_00952 | 1.2337E-28 | 3.72 | 30S ribosomal protein S12 |
| D7S_00348 | 2.27728E-29 | 3.72 | hypothetical protein |
| D7S_01083 | 1.30959E-28 | 3.68 | 50S ribosomal protein L28 |
| D7S_02046 | 1.48174E-31 | 3.67 | fructose-1,6-bisphosphatase |
| D7S_02229 | 2.14274E-10 | 3.66 | ABC transporter permease |
| D7S_00415 | 7.2175E-18 | 3.62 | hypothetical protein |
| D7S_02432 | 0.000685656 | 3.61 | threonyl-tRNA synthetase |
| D7S_00683 | 1.18366E-29 | 3.60 | hypothetical protein |
| D7S_02074 | 1.48458E-36 | 3.60 | outer membrane protein 39 |
| D7S_00780 | 1.29288E-32 | 3.59 | 30S ribosomal protein S11 |
| D7S_02096 | 3.68603E-16 | 3.59 | glycoside hydrolase, family 19, putative |
| D7S_00835 | 1.94437E-32 | 3.55 | 30S ribosomal protein S16 |
| D7S_01832 | 7.35011E-19 | 3.54 | hypothetical protein |
| D7S_00791 | 5.59402E-35 | 3.54 | 50S ribosomal protein L24 |
| D7S_02097 | 3.8476E-08 | 3.50 | glycoside hydrolase, family 19, putative |
| D7S_01071 | 2.85102E-24 | 3.50 | AcrA protein |
| D7S_02187 | 0.001890773 | 3.49 | gluconate permease |
| D7S_01487 | 8.89363E-29 | 3.49 | UDP-3-O-acyl N-acetylglucosamine deacetylase |
| D7S_02425 | 9.32036E-24 | 3.48 | NAD dehydrogenase |
| D7S_02329 | 4.32774E-06 | 3.48 | para-aminobenzoate synthase glutamine |
| D7S_00587 | 6.33237E-22 | 3.46 | hypothetical protein |
| D7S_02099 | 1.20821E-09 | 3.46 | PTS system galactitol-specific transporter |
| D7S_00626 | 2.16108E-16 | 3.46 | biopolymer transport protein |
| D7S_00468 | 1.84559E-14 | 3.45 | YadA-like protein |
| D7S_02365 | 2.01846E-29 | 3.43 | ribonuclease G |
| D7S_00271 | 0.010973151 | 3.43 | TRNA/rRNA methyltransferase |
| D7S_02340 | 7.64678E-28 | 3.43 | translation initiation factor IF-3 |
| D7S_00628 | 5.06781E-30 | 3.42 | tol-pal system-associated acyl-CoA thioesterase |
| D7S_01718 | 0.001530674 | 3.42 | hypothetical protein |
| D7S_00993 | 5.3788E-10 | 3.41 | LysR protein |
| D7S_01252 | 0.01258628 | 3.41 | hypothetical protein |
| D7S_01595 | 2.55601E-29 | 3.39 | YihD like protein |
| D7S_01002 | 4.43692E-23 | 3.37 | SEC-C motif domain-containing protein |
| D7S_01815 | 1.24684E-27 | 3.35 | 50S ribosomal protein L4 |
| D7S_00759 | 1.9291E-10 | 3.35 | putative FHA domain protein |
| D7S_00054 | 6.16428E-14 | 3.34 | AcrR protein |
| D7S_00279 | 0.025963573 | 3.33 | hypothetical protein |
| D7S_01821 | 1.42037E-30 | 3.31 | 50S ribosomal protein L16 |
| D7S_01018 | 1.30125E-24 | 3.30 | carbon storage regulator |
| D7S_00782 | 7.01281E-30 | 3.29 | 50S ribosomal protein L36 |
| D7S_00277 | 0.001134413 | 3.28 | hypothetical protein |
| D7S_01651 | 4.11237E-15 | 3.27 | hypothetical protein |
| D7S_02296 | 8.11646E-20 | 3.26 | cytolethal distending toxin protein C |
| D7S_00358 | 8.91728E-15 | 3.24 | hypothetical protein |
| D7S_00107 | 6.96452E-22 | 3.23 | putative plasmid stability protein StbD, |
| D7S_01022 | 0.00157807 | 3.22 | hypothetical protein |
| D7S_02076 | 1.61228E-25 | 3.22 | pemk-like protein 2 |
| D7S_02241 | 9.57566E-25 | 3.21 | hypothetical protein |
| D7S_02007 | 4.93034E-24 | 3.17 | succinyl-diaminopimelate desuccinylase |
| D7S_02423 | 6.81418E-29 | 3.14 | HflC protein |
| D7S_00526 | 1.37143E-12 | 3.13 | S-adenosylmethionine--tRNA |
| D7S_00473 | 3.22615E-25 | 3.11 | GMP synthase |
| D7S_00192 | 1.6343E-19 | 3.11 | putative transcriptional regulator |
| D7S_02304 | 0.041464558 | 3.11 | hypothetical protein |
| D7S_00786 | 4.7512E-27 | 3.11 | 50S ribosomal protein L18 |
| D7S_00803 | 1.75421E-08 | 3.11 | hypothetical protein |
| D7S_01070 | 1.02013E-23 | 3.09 | AcrA protein |
| D7S_01427 | 1.73322E-25 | 3.07 | protein RecA |
| D7S_00756 | 2.30842E-16 | 3.07 | hypothetical protein |
| D7S_00777 | 2.78175E-27 | 3.07 | 50S ribosomal protein L17 |
| D7S_00283 | 0.000516983 | 3.06 | tRNA/rRNA methyltransferase |
| D7S_00124 | 1.16677E-06 | 3.06 | tRNA pseudouridine synthase C |
| D7S_00825 | 0.022585298 | 3.05 | hypothetical protein |
| D7S_00353 | 5.22395E-15 | 3.04 | protein YhbC |
| D7S_01549 | 4.23936E-19 | 3.04 | hypothetical protein |
| D7S_01669 | 1.82828E-23 | 3.04 | lipoprotein |
| D7S_01385 | 7.47182E-24 | 3.04 | outer membrane protein 18/16 |
| D7S_01779 | 9.11684E-11 | 3.03 | 4-hydroxybenzoate |
| D7S_01562 | 2.60595E-14 | 3.00 | nucleoside diphosphate kinase |
| D7S_00455 | 5.17145E-07 | 3.00 | glycerol-3-phosphate dehydrogenase |
| D7S_01776 | 8.46023E-22 | 2.94 | DNA-directed RNA polymerase subunit omega |
| D7S_01323 | 1.44252E-06 | 2.92 | quinol dehydrogenase periplasmic component |
| D7S_02026 | 2.68071E-14 | 2.90 | ABC transporter permease |
| D7S_00262 | 8.17579E-15 | 2.89 | peptide chain release factor 2 |
| D7S_02237 | 1.09252E-21 | 2.88 | 3-deoxy-7-phosphoheptulonate synthase |
| D7S_00698 | 3.58954E-05 | 2.88 | integrase catalytic subunit |
| D7S_00838 | 5.3894E-22 | 2.87 | 50S ribosomal protein L19 |
| D7S_01453 | 4.86837E-18 | 2.86 | type II/IV secretion system secretin RcpA/CpaC |
| D7S_00753 | 8.91927E-18 | 2.82 | peptidyl-prolyl cis-trans isomerase B |
| D7S_02394 | 1.30587E-16 | 2.79 | nucleic acid-binding protein |
| D7S_00644 | 2.64918E-06 | 2.79 | ribonuclease T |
| D7S_00064 | 4.83842E-21 | 2.77 | ferritin like protein-2 |
| D7S_00785 | 3.4811E-22 | 2.77 | 30S ribosomal protein S5 |
| D7S_01640 | 3.80961E-16 | 2.77 | D,D-heptose 1,7-bisphosphate phosphatase |
| D7S_00778 | 1.47995E-24 | 2.77 | DNA-directed RNA polymerase subunit alpha |
| D7S_02292 | 3.1613E-20 | 2.76 | transcriptional modulator of MazE/toxin MazF |
| D7S_01817 | 2.44941E-22 | 2.76 | 50S ribosomal protein L2 |
| D7S_01214 | 1.06785E-21 | 2.76 | peroxiredoxin like protein |
| D7S_00799 | 5.50839E-15 | 2.75 | WD-40 repeat containing protein, putative |
| D7S_00953 | 1.0416E-20 | 2.73 | 30S ribosomal protein S7 |
| D7S_00471 | 3.59414E-06 | 2.73 | putative RelB protein |
| D7S_00212 | 1.56668E-20 | 2.72 | NlpD protein |
| D7S_01714 | 9.78974E-12 | 2.72 | WD-40 repeat containing protein, putative |
| D7S_01181 | 1.09976E-23 | 2.72 | putative metal/nucleic acid-binding protein |
| D7S_01122 | 2.93258E-16 | 2.71 | translocase |
| D7S_02341 | 3.84459E-22 | 2.71 | hypothetical protein |
| D7S_00280 | 0.033283786 | 2.70 | hypothetical protein |
| D7S_01891 | 1.02904E-06 | 2.67 | cfr family radical SAM enzyme |
| D7S_01215 | 7.00371E-16 | 2.66 | DNA-binding transcriptional regulator OxyR |
| D7S_00783 | 1.19408E-22 | 2.66 | preprotein translocase subunit SecY |
| D7S_00781 | 1.83152E-20 | 2.66 | 30S ribosomal protein S13 |
| D7S_02190 | 2.61341E-05 | 2.65 | hypothetical protein |
| D7S_02016 | 1.74857E-08 | 2.64 | protein YegH |
| D7S_00534 | 0.029542636 | 2.64 | hypothetical protein |
| D7S_01853 | 1.17679E-12 | 2.64 | hypothetical protein |
| D7S_02045 | 9.28449E-18 | 2.63 | UDP-N-acetylmuramate:L-alanyl-gamma-D-glutamyl--meso-diaminopimelate ligase |
| D7S_01554 | 6.85699E-06 | 2.62 | citrate lyase subunit gamma |
| D7S_01723 | 3.3246E-05 | 2.62 | putative fimbrial subunit PilA |
| D7S_00462 | 1.2343E-19 | 2.62 | peptide methionine sulfoxide reductase |
| D7S_00583 | 1.99166E-11 | 2.61 | putative L-ascorbate 6-phosphate lactonase |
| D7S_00488 | 4.83829E-17 | 2.60 | phosphate transport regulator |
| D7S_00111 | 7.43655E-18 | 2.59 | 30S ribosomal protein S9 |
| D7S_00497 | 3.71688E-16 | 2.59 | L-lactate transport |
| D7S_01314 | 6.48311E-20 | 2.59 | alternative sigma factor RpoH |
| D7S_01813 | 4.26873E-19 | 2.59 | 30S ribosomal protein S10 |
| D7S_00058 | 3.03628E-09 | 2.59 | fumarate hydratase |
| D7S_01816 | 8.64537E-21 | 2.58 | 50S ribosomal protein L23 |
| D7S_01966 | 4.60211E-21 | 2.57 | GTP-binding protein LepA |
| D7S_00605 | 2.00812E-13 | 2.57 | hemolysin-activating lysine-acyltransferase |
| D7S_00682 | 5.42704E-15 | 2.57 | ribonuclease E |
| D7S_01596 | 1.51683E-15 | 2.56 | thiol:disulfide interchange protein DsbA |
| D7S_01008 | 7.01917E-16 | 2.56 | OmpR protein |
| D7S_00906 | 3.15094E-06 | 2.54 | hypothetical protein |
| D7S_00922 | 5.6862E-07 | 2.54 | lipid-A-disaccharide synthase |
| D7S_01907 | 3.8119E-20 | 2.54 | superoxide dismutase |
| D7S_00911 | 0.002288579 | 2.53 | hypothetical protein |
| D7S_00788 | 3.31985E-20 | 2.53 | 30S ribosomal protein S8 |
| D7S_00147 | 1.38076E-19 | 2.53 | membrane protein |
| D7S_01106 | 4.08145E-07 | 2.53 | Integral membrane protein |
| D7S_00452 | 2.01339E-05 | 2.53 | fmn-dependent NADH-azoreductase |
| D7S_01238 | 5.36218E-19 | 2.53 | ATP synthase F0 subunit B |
| D7S_01928 | 0.016669352 | 2.51 | hypothetical protein |
| D7S_01457 | 8.72824E-18 | 2.51 | fimbrial protein Flp precursor |
| D7S_01708 | 1.92906E-17 | 2.50 | 50S ribosomal protein L10 |
| D7S_02011 | 1.14023E-12 | 2.50 | dihydrodipicolinate synthase |
| D7S_00267 | 0.008291791 | 2.49 | hypothetical protein |
| D7S_01777 | 7.26693E-16 | 2.49 | GTP pyrophosphokinase |
| D7S_00706 | 2.44826E-16 | 2.47 | iron-sulfur cluster assembly protein IscA |
| D7S_00553 | 2.21446E-06 | 2.46 | exoribonuclease II |
| D7S_01985 | 1.50919E-19 | 2.46 | hypothetical protein |
| D7S_01467 | 3.68896E-11 | 2.46 | glutamine transport ATP-binding protein GlnQ |
| D7S_01564 | 7.9519E-07 | 2.46 | putative transposase |
| D7S_00549 | 0.000228924 | 2.45 | queuosine biosynthesis protein QueD |
| D7S_02313 | 3.12233E-17 | 2.45 | phosphocarrier protein HPr |
| D7S_00790 | 7.84965E-18 | 2.45 | 50S ribosomal protein L5 |
| D7S_00680 | 0.006964199 | 2.42 | bifunctional |
| D7S_00687 | 6.56601E-17 | 2.42 | cystathionine beta-lyase |
| D7S_02252 | 2.87397E-20 | 2.41 | 5-methylaminomethyl-2-thiouridine |
| D7S_01518 | 2.86435E-19 | 2.41 | hypothetical protein |
| D7S_00171 | 3.5745E-16 | 2.40 | integration host factor subunit beta |
| D7S_00162 | 2.64774E-06 | 2.39 | esterase YbfF |
| D7S_01737 | 9.52656E-15 | 2.39 | hypothetical protein |
| D7S_02029 | 1.3626E-12 | 2.38 | hypothetical protein |
| D7S_00329 | 0.007846751 | 2.38 | DNA internalization-related competence protein |
| D7S_01272 | 5.50304E-11 | 2.38 | ABC transporter permease |
| D7S_01324 | 0.028923655 | 2.37 | ferredoxin-type protein NapH |
| D7S_02019 | 6.3538E-08 | 2.34 | FadL protein |
| D7S_01728 | 7.2905E-17 | 2.34 | lipoprotein NlpI |
| D7S_02286 | 5.07121E-16 | 2.34 | hypothetical protein |
| D7S_00309 | 9.59086E-09 | 2.33 | hypothetical protein |
| D7S_00371 | 1.12562E-11 | 2.33 | undecaprenyl-phosphate |
| D7S_00143 | 4.4049E-12 | 2.33 | formyltetrahydrofolate deformylase |
| D7S_00550 | 0.019615495 | 2.33 | NrdG protein |
| D7S_02390 | 5.27075E-13 | 2.33 | dihydrodipicolinate reductase |
| D7S_00122 | 0.000821924 | 2.32 | hypothetical protein |
| D7S_02192 | 6.01413E-05 | 2.32 | toxin |
| D7S_00684 | 1.17815E-09 | 2.32 | ribosomal large subunit pseudouridine synthase |
| D7S_01015 | 0.000110769 | 2.32 | nucleoside triphosphate pyrophosphohydrolase |
| D7S_01218 | 9.02718E-14 | 2.30 | ABC superfamily ATP binding cassette |
| D7S_01986 | 1.50819E-14 | 2.30 | helix-turn-helix containing protein |
| D7S_01048 | 3.19874E-07 | 2.30 | hypothetical protein |
| D7S_01201 | 0.005004732 | 2.30 | hypothetical protein |
| D7S_00328 | 6.84172E-14 | 2.29 | RNA polymerase-binding protein DksA |
| D7S_01183 | 2.50166E-13 | 2.29 | fatty acid/phospholipid synthesis protein PlsX |
| D7S_00373 | 9.28494E-07 | 2.29 | protein-P-II uridylyltransferase |
| D7S_01725 | 0.038204343 | 2.29 | hypothetical protein |
| D7S_00273 | 0.006639869 | 2.28 | tRNA/rRNA methyltransferase |
| D7S_01814 | 3.53626E-15 | 2.28 | 50S ribosomal protein L3 |
| D7S_01146 | 6.13479E-13 | 2.28 | hemolysin |
| D7S_01879 | 5.94437E-15 | 2.27 | ATP-dependent protease La |
| D7S_02119 | 4.81956E-07 | 2.27 | hypothetical protein |
| D7S_01200 | 8.36995E-14 | 2.26 | lipoic acid synthetase |
| D7S_00303 | 1.19777E-11 | 2.25 | HTH-type transcriptional regulator CysB |
| D7S_01320 | 0.000863132 | 2.25 | NapD protein |
| D7S_00393 | 5.50452E-12 | 2.24 | putative glutaredoxin-like protein |
| D7S_00865 | 1.46505E-13 | 2.24 | phosphatidylserine synthase |
| D7S_00327 | 6.88908E-06 | 2.24 | polyA polymerase |
| D7S_00932 | 2.46261E-15 | 2.24 | ribosome recycling factor |
| D7S_02366 | 3.33647E-15 | 2.23 | plasmid maintenance system killer protein |
| D7S_00541 | 0.000148683 | 2.23 | methylmalonate-semialdehyde dehydrogenase |
| D7S_00479 | 3.92766E-05 | 2.22 | aminopeptidase C |
| D7S_00627 | 5.35836E-16 | 2.22 | protein TolQ |
| D7S_01166 | 0.008714588 | 2.20 | phospholipase D/Transphosphatidylase |
| D7S_02270 | 2.36263E-10 | 2.19 | GTP-binding proten HflX |
| D7S_01187 | 6.99593E-15 | 2.19 | acyl carrier protein |
| D7S_00173 | 6.74395E-14 | 2.18 | cytidylate kinase |
| D7S_01823 | 3.52346E-15 | 2.17 | 30S ribosomal protein S17 |
| D7S_01581 | 0.000646513 | 2.17 | transposase |
| D7S_01722 | 0.021412348 | 2.16 | type IV pilus assembly protein |
| D7S_00208 | 2.62784E-08 | 2.16 | ABC transport system periplasmic protein |
| D7S_02369 | 0.000903609 | 2.15 | integrase catalytic subunit |
| D7S_01768 | 6.04185E-11 | 2.15 | hypothetical protein |
| D7S_00725 | 1.60134E-09 | 2.15 | dinucleoside polyphosphate hydrolase |
| D7S_02186 | 0.004957611 | 2.14 | shikimate kinase |
| D7S_00449 | 1.41012E-13 | 2.14 | glutathione S-transferase |
| D7S_02228 | 0.004957611 | 2.14 | TonB-dependent siderophore receptor |
| D7S_01520 | 1.72328E-13 | 2.14 | monofunctional biosynthetic peptidoglycan |
| D7S_02343 | 0.001633359 | 2.13 | aerobic C4-dicarboxylate transport protein |
| D7S_02385 | 4.12487E-15 | 2.13 | 6,7-dimethyl-8-ribityllumazine synthase |
| D7S_02342 | 4.55034E-12 | 2.13 | 50S ribosomal protein L20 |
| D7S_02120 | 1.03483E-11 | 2.13 | hypothetical protein |
| D7S_02445 | 0.005644555 | 2.12 | WD-40 repeat containing protein, putative |
| D7S_01001 | 4.22028E-09 | 2.12 | uridine phosphorylase |
| D7S_01519 | 4.35296E-12 | 2.11 | hypothetical protein |
| D7S_02422 | 0.001452649 | 2.11 | putative glucanotransferase |
| D7S_01277 | 1.07046E-08 | 2.10 | hypothetical protein |
| D7S_00971 | 1.96746E-12 | 2.10 | RNA polymerase sigma-70 factor family protein |
| D7S_00573 | 0.000400903 | 2.10 | LacZ protein |
| D7S_00284 | 2.23842E-12 | 2.10 | Tra5 protein low-quality sequence region |
| D7S_00787 | 7.42556E-13 | 2.09 | 50S ribosomal protein L6 |
| D7S_01081 | 2.07484E-09 | 2.09 | heptosyltransferase family |
| D7S_02291 | 2.29922E-13 | 2.09 | hypothetical protein |
| D7S_01915 | 8.03832E-14 | 2.09 | oxido-reductase |
| D7S_01916 | 4.71058E-14 | 2.08 | sodium/proton antiporter |
| D7S_00167 | 5.85316E-13 | 2.08 | translation initiation factor Sui1 |
| D7S_01872 | 3.03876E-08 | 2.07 | alpha-glucosidase |
| D7S_00112 | 3.26242E-12 | 2.07 | 50S ribosomal protein L13 |
| D7S_01960 | 4.24136E-05 | 2.07 | ribonuclease P protein component |
| D7S_02015 | 3.74049E-12 | 2.07 | glutamine synthetase, type I |
| D7S_00649 | 4.85743E-06 | 2.06 | outer membrane protein 64 |
| D7S_01905 | 1.78013E-05 | 2.05 | hypothetical protein |
| D7S_00164 | 4.37492E-12 | 2.05 | ferric uptake regulation protein |
| D7S_01707 | 1.93847E-10 | 2.04 | 50S ribosomal protein L7/L12 |
| D7S_01239 | 4.54862E-12 | 2.04 | ATP synthase F1 subunit delta |
| D7S_00220 | 5.89431E-05 | 2.03 | electron transport complex protein RnfD |
| D7S_01007 | 4.1705E-06 | 2.03 | sensor protein CpxA |
| D7S_01424 | 5.47241E-07 | 2.03 | hypothetical protein |
| D7S_02155 | 8.60863E-11 | 2.02 | twin-arginine translocation pathway signal |
| D7S_00991 | 1.98609E-11 | 2.01 | D-fructose-6-phosphate amidotransferase |
| D7S_00846 | 0.000513251 | 2.01 | glycerophosphoryl diester phosphodiesterase |
| D7S_02157 | 9.65486E-05 | 2.01 | hypothetical protein |
| D7S_00443 | 5.65923E-10 | 2.00 | high-affinity nickel-transporter |
| D7S_01820 | 2.61979E-10 | 2.00 | 30S ribosomal protein S3 |
| D7S_01967 | 7.41029E-12 | 1.99 | autonomous glycyl radical cofactor GrcA |
| D7S_01247 | 1.25408E-11 | 1.99 | UDP-N-acetylglucosamine |
| D7S_00703 | 2.40552E-09 | 1.99 | iron-sulfur cluster assembly transcription |
| D7S_00198 | 0.008642061 | 1.99 | L-lactate dehydrogenase |
| D7S_01186 | 6.00628E-12 | 1.99 | 3-ketoacyl-ACP reductase |
| D7S_00460 | 9.00844E-08 | 1.98 | OsmY protein |
| D7S_01886 | 3.33147E-07 | 1.98 | hypothetical protein |
| D7S_01826 | 3.46137E-05 | 1.97 | transposase |
| D7S_00983 | 4.37349E-09 | 1.97 | phosphoenolpyruvate carboxykinase |
| D7S_00150 | 7.2407E-07 | 1.96 | hypothetical protein |
| D7S_01913 | 1.13028E-09 | 1.95 | ArtI protein |
| D7S_00357 | 0.000139662 | 1.95 | hypothetical protein |
| D7S_02277 | 0.006761654 | 1.94 | putative transmembrane protein |
| D7S_00472 | 5.53746E-05 | 1.94 | addiction module antitoxin |
| D7S_02309 | 1.06927E-08 | 1.94 | lipoprotein |
| D7S_00044 | 3.50441E-05 | 1.94 | heat shock protein HtpX |
| D7S_00464 | 8.44921E-08 | 1.93 | phosphoribosylformylglycinamidine synthase |
| D7S_01847 | 5.18035E-05 | 1.93 | phosphoserine phosphatase |
| D7S_00312 | 3.55276E-10 | 1.93 | dihydrolipoamide acetyltransferase component of |
| D7S_02434 | 0.009791438 | 1.93 | TonB-dependent receptor |
| D7S_01565 | 2.87006E-07 | 1.92 | XRE family transcriptional regulator |
| D7S_02032 | 9.6694E-12 | 1.92 | nitrogen regulatory IIA protein |
| D7S_00988 | 1.0775E-09 | 1.92 | D-fructose-6-phosphate amidotransferase |
| D7S_01524 | 1.41479E-07 | 1.92 | acyl-CoA thioester hydrolase YciA |
| D7S_02142 | 1.37626E-10 | 1.91 | seryl-tRNA synthetase |
| D7S_01400 | 1.05281E-09 | 1.91 | transposase |
| D7S_00100 | 2.01163E-06 | 1.91 | death-on-curing protein |
| D7S_02380 | 0.001131086 | 1.91 | hypothetical protein |
| D7S_01988 | 0.003933334 | 1.91 | tyrosine phenol-lyase |
| D7S_00592 | 3.29248E-05 | 1.91 | hypothetical protein |
| D7S_00590 | 0.023642808 | 1.90 | glycoside hydrolase, family 19, putative |
| D7S_02027 | 1.4431E-05 | 1.89 | ABC transporter ATPase |
| D7S_01846 | 8.22008E-07 | 1.89 | nucleotide-binding protein |
| D7S_01128 | 0.000250732 | 1.89 | diadenosine tetraphosphatase |
| D7S_00826 | 7.44417E-05 | 1.89 | hypothetical protein |
| D7S_00888 | 1.78702E-07 | 1.88 | dna-3-methyladenine glycosyllase 1 |
| D7S_01092 | 3.83945E-10 | 1.87 | phosphopyruvate hydratase |
| D7S_00849 | 1.44494E-07 | 1.86 | 30S ribosomal protein S18 |
| D7S_00965 | 5.68263E-10 | 1.86 | LysR family transcriptional regulator |
| D7S_01688 | 0.015072154 | 1.85 | transposase |
| D7S_02189 | 0.001604251 | 1.85 | DNA ligase |
| D7S_02333 | 4.70749E-09 | 1.85 | threonyl-tRNA synthetase |
| D7S_01871 | 0.009791342 | 1.85 | TRAP dicarboxylate transporter subunit DctM |
| D7S_00341 | 0.002452375 | 1.84 | hypothetical protein |
| D7S_02352 | 1.04934E-07 | 1.84 | acetyl-CoA carboxylase, carboxyl transferase |
| D7S_00692 | 0.000148293 | 1.84 | integrase catalytic subunit |
| D7S_02240 | 8.17955E-07 | 1.84 | septum site-determining protein MinC |
| D7S_01372 | 4.85699E-05 | 1.84 | ATPase family protein |
| D7S_01079 | 0.044268105 | 1.83 | putative D-glycero-D-manno-heptosyl transferase |
| D7S_01178 | 2.5141E-06 | 1.83 | dithiobiotin synthetase |
| D7S_01391 | 4.96858E-09 | 1.82 | S-ribosylhomocysteinase |
| D7S_02288 | 6.27115E-07 | 1.82 | hypothetical protein |
| D7S_00876 | 5.49282E-09 | 1.81 | translation elongation factor Tu |
| D7S_00028 | 3.16761E-05 | 1.81 | transferrin-binding protein 1 |
| D7S_00691 | 1.14621E-05 | 1.80 | hypothetical protein |
| D7S_01207 | 1.44349E-05 | 1.80 | L-cystine import ATP-binding protein TcyN |
| D7S_02361 | 2.25359E-06 | 1.79 | alpha/beta superfamily hydrolase |
| D7S_02278 | 0.013905966 | 1.79 | putative phage glucose translocase |
| D7S_01965 | 6.79567E-10 | 1.79 | signal peptidase I |
| D7S_00480 | 9.22417E-08 | 1.79 | UDP-glucose 4-epimerase |
| D7S_02039 | 4.0043E-07 | 1.79 | hypothetical protein |
| D7S_01849 | 6.64104E-08 | 1.79 | 30S ribosomal protein S20 |
| D7S_00630 | 1.11266E-07 | 1.77 | cytochrome D ubiquinol oxidase subunit 1 |
| D7S_02280 | 1.19245E-08 | 1.77 | glycosyll transferase, family 2 |
| D7S_01094 | 5.8811E-09 | 1.77 | aminopeptidase B |
| D7S_01512 | 5.25198E-07 | 1.77 | hypothetical protein |
| D7S_00774 | 0.000729719 | 1.77 | murein transglycosyllase C |
| D7S_00193 | 0.001277434 | 1.76 | diaminopimelate decarboxylase |
| D7S_01686 | 0.02970316 | 1.76 | Tra5 protein |
| D7S_01484 | 1.40049E-07 | 1.76 | D-alanine--D-alanine ligase |
| D7S_00623 | 9.90798E-09 | 1.75 | peptidoglycan-associated lipoprotein |
| D7S_01673 | 8.77979E-08 | 1.75 | sodium/panthothenate symporter |
| D7S_02033 | 2.76142E-08 | 1.75 | ATPase |
| D7S_01011 | 0.000475485 | 1.75 | hypothetical protein |
| D7S_02346 | 0.037220158 | 1.75 | tagatose-bisphosphate aldolase noncatalytic |
| D7S_01353 | 5.44491E-06 | 1.75 | hypothetical protein |
| D7S_01709 | 3.15865E-07 | 1.74 | 50S ribosomal protein L1 |
| D7S_00461 | 1.4751E-07 | 1.74 | carbonate dehydratase |
| D7S_01148 | 0.000103048 | 1.74 | TonB-system energizer ExbB |
| D7S_00977 | 4.69675E-08 | 1.74 | porphobilinogen deaminase |
| D7S_00085 | 3.65355E-08 | 1.74 | glycoside hydrolase, family 19, putative |
| D7S_01039 | 0.015236107 | 1.74 | BolA protein |
| D7S_01910 | 2.18564E-06 | 1.73 | XkdP protein |
| D7S_01645 | 1.12731E-07 | 1.73 | hypothetical protein |
| D7S_02118 | 0.018013499 | 1.72 | putative DEAD/DEAH box helicase |
| D7S_00421 | 0.014185284 | 1.72 | D-alanyl-D-alanine carboxypeptidase |
| D7S_01271 | 0.010737244 | 1.71 | hypothetical protein |
| D7S_00986 | 0.003867757 | 1.71 | NADH pyrophosphatase |
| D7S_01594 | 2.10484E-05 | 1.71 | molybdopterin-guanine dinucleotide biosynthesis |
| D7S_00910 | 0.010362024 | 1.71 | putative FHA domain protein |
| D7S_00954 | 1.83821E-07 | 1.71 | elongation factor G |
| D7S_00396 | 0.001252013 | 1.71 | esterase YdiI |
| D7S_01468 | 1.2377E-05 | 1.70 | arginine-binding periplasmic protein 1 |
| D7S_00704 | 3.87973E-06 | 1.70 | cysteine desulfurase IscS |
| D7S_02234 | 1.52769E-06 | 1.69 | outer membrane lipoprotein pcp |
| D7S_00776 | 3.70463E-08 | 1.69 | phosphoglycerate kinase |
| D7S_00530 | 4.30159E-05 | 1.69 | preprotein translocase subunit YajC |
| D7S_00295 | 9.003E-05 | 1.68 | hypothetical protein |
| D7S_01254 | 0.033255127 | 1.68 | hypothetical protein |
| D7S_00773 | 6.12316E-05 | 1.68 | nicotinamide-nucleotide adenylyltransferase |
| D7S_00659 | 2.94105E-05 | 1.68 | heptaprenyl diphosphate synthase component II |
| D7S_01685 | 5.01151E-06 | 1.68 | ISSod1, transposase OrfB |
| D7S_01367 | 3.28211E-06 | 1.67 | IscR-regulated protein YhgI |
| D7S_02008 | 7.39901E-05 | 1.67 | glycine betaine transport ATP-binding protein |
| D7S_01265 | 0.000337244 | 1.67 | Rhs element Vgr protein |
| D7S_02271 | 0.01628646 | 1.66 | GTP-binding protein HflX |
| D7S_00278 | 0.000966688 | 1.66 | tRNA/rRNA methyltransferase |
| D7S_01955 | 0.000129364 | 1.65 | methyltransferase domain-containing protein |
| D7S_00686 | 1.72051E-06 | 1.65 | cysteinyl-tRNA synthetase |
| D7S_00414 | 0.001783411 | 1.65 | PlyM26 |
| D7S_00923 | 2.62487E-07 | 1.65 | UDP-N-acetylglucosamine acyltransferase |
| D7S_02406 | 8.22838E-06 | 1.65 | maltose/maltodextrin import ATP-binding protein |
| D7S_01785 | 4.91693E-07 | 1.65 | peptide deformylase |
| D7S_00180 | 5.67938E-07 | 1.65 | 5-methyltetrahydropteroyltriglutamate/homocysteine S-methyltransferase |
| D7S_02389 | 0.030054467 | 1.64 | threonine efflux protein |
| D7S_01188 | 0.020913932 | 1.64 | hypothetical protein |
| D7S_01276 | 0.001402849 | 1.64 | hypothetical protein |
| D7S_02424 | 3.00513E-05 | 1.64 | regulator of membrane protease activity |
| D7S_02022 | 2.48285E-05 | 1.64 | BolA protein |
| D7S_00928 | 1.95039E-07 | 1.64 | RIP metalloprotease RseP |
| D7S_00885 | 0.001716984 | 1.64 | lipoprotein |
| D7S_02287 | 3.18959E-06 | 1.64 | hypothetical protein |
| D7S_00636 | 3.91764E-06 | 1.63 | dATP pyrophosphohydrolase |
| D7S_01429 | 0.002803989 | 1.63 | endoribonuclease L-PSP |
| D7S_01592 | 0.000533285 | 1.63 | multiphosphoryl transfer protein |
| D7S_02130 | 1.92123E-06 | 1.63 | hypothetical protein |
| D7S_01738 | 0.031854147 | 1.63 | transporter, NadC family |
| D7S_01774 | 2.03054E-06 | 1.63 | glyceraldehyde-3-phosphate dehydrogenase, type |
| D7S_00822 | 0.000661622 | 1.62 | NADPH-flavin oxidoreductase |
| D7S_00315 | 5.62478E-06 | 1.62 | tellurite resistance protein TehB |
| D7S_02220 | 7.70099E-07 | 1.62 | GTP cyclohydrolase I |
| D7S_02376 | 0.000698609 | 1.61 | lipoprotein releasing system, transmembrane |
| D7S_02150 | 0.000957696 | 1.61 | PqiA family integral membrane protein |
| D7S_02098 | 0.00016664 | 1.61 | hypothetical protein |
| D7S_00163 | 4.24689E-06 | 1.60 | flavodoxin |
| D7S_00060 | 0.00087657 | 1.60 | universal stress protein UspE |
| D7S_02012 | 0.000179876 | 1.60 | peroxiredoxin Q |
| D7S_01559 | 6.93869E-06 | 1.59 | translation initiation factor IF-1 |
| D7S_01646 | 0.002541895 | 1.59 | transposase |
| D7S_00098 | 0.000125561 | 1.59 | hypothetical protein |
| D7S_01964 | 1.47087E-05 | 1.59 | ribonuclease III |
| D7S_01110 | 0.010882709 | 1.59 | SecA-related protein |
| D7S_01345 | 0.003252354 | 1.59 | glycosyll transferase, family 2 |
| D7S_00082 | 0.004282006 | 1.59 | 4_alpha-glucanotransferase |
| D7S_01090 | 0.000125688 | 1.58 | deoxyuridine 5'-triphosphate |
| D7S_00974 | 3.03377E-05 | 1.58 | HemY protein |
| D7S_01469 | 1.84996E-05 | 1.58 | histidine transport system permease HisQ |
| D7S_01710 | 4.60626E-05 | 1.58 | 50S ribosomal protein L11 |
| D7S_00710 | 5.73779E-05 | 1.58 | FeS assembly protein IscX |
| D7S_00661 | 2.24792E-06 | 1.58 | 50S ribosomal protein L27 |
| D7S_01237 | 4.23468E-05 | 1.58 | F0F1 ATP synthase subunit C |
| D7S_00170 | 0.007683569 | 1.58 | 30S ribosomal protein S1 |
| D7S_00696 | 0.006172768 | 1.58 | FHA domain-containing protein |
| D7S_00715 | 0.025104749 | 1.57 | hypothetical protein |
| D7S_00832 | 1.43463E-05 | 1.57 | Z-ring-associated protein ZapA |
| D7S_01859 | 0.007091504 | 1.57 | protein YbjQ |
| D7S_01987 | 4.2352E-06 | 1.57 | tyrosine-specific transporter |
| D7S_00420 | 0.018720536 | 1.56 | hypothetical protein |
| D7S_01179 | 3.83231E-05 | 1.56 | biotin biosynthesis protein BioC |
| D7S_01354 | 0.000692102 | 1.56 | lipoprotein |
| D7S_00046 | 8.16243E-05 | 1.55 | phenylalanyl-tRNA synthetase subunit beta |
| D7S_02395 | 1.65371E-05 | 1.55 | hypothetical protein |
| D7S_02386 | 1.26051E-05 | 1.55 | transcription antitermination factor NusB |
| D7S_01473 | 2.2501E-05 | 1.55 | S-adenosyl-methyltransferase MraW |
| D7S_02191 | 0.027724046 | 1.55 | molybdate ABC transporter permease |
| D7S_01065 | 0.000327015 | 1.55 | GTP pyrophosphokinase |
| D7S_01227 | 3.74643E-05 | 1.55 | transcriptional regulator |
| D7S_01690 | 0.00011896 | 1.55 | amino acid ligase |
| D7S_01284 | 0.000141986 | 1.54 | aspartate ammonia-lyase |
| D7S_00660 | 2.83884E-05 | 1.54 | 50S ribosomal protein L21 |
| D7S_00637 | 0.004989022 | 1.54 | dATP pyrophosphohydrolase |
| D7S_02426 | 1.87372E-05 | 1.54 | TorD protein |
| D7S_00511 | 0.000245158 | 1.54 | putative relE protein |
| D7S_00023 | 0.009345797 | 1.54 | ribonucleoside-diphosphate reductase subunit |
| D7S_01452 | 0.002156178 | 1.54 | RcpB protein |
| D7S_00913 | 0.01714635 | 1.54 | hypothetical protein |
| D7S_00565 | 0.003459672 | 1.54 | valyl-tRNA synthetase |
| D7S_00700 | 0.010926853 | 1.53 | hypothetical protein |
| D7S_00894 | 0.006000384 | 1.53 | RseC protein |
| D7S_00545 | 0.005621656 | 1.53 | CRISPR-associated protein |
| D7S_00652 | 0.007261664 | 1.53 | hypothetical protein |
| D7S_02021 | 1.75224E-05 | 1.53 | UDP-N-acetylglucosamine |
| D7S_01998 | 2.73862E-05 | 1.53 | HflC protein |
| D7S_00086 | 0.000229981 | 1.52 | ABC transporter ATP-binding protein uup |
| D7S_01282 | 2.76079E-05 | 1.52 | chaperonin GroS |
| D7S_00261 | 7.55262E-06 | 1.52 | thiol:disulfide interchange protein DsbC |
| D7S_01482 | 6.8388E-06 | 1.52 | UDP-N-acetylmuramate--L-alanine ligase |
| D7S_01093 | 0.000182803 | 1.52 | cyclic nucleotide-binding domain-containing |
| D7S_00970 | 0.000675966 | 1.52 | dsDNA-mimic protein |
| D7S_01522 | 0.004467298 | 1.51 | intracellular septation protein A |
| D7S_02013 | 5.39573E-05 | 1.51 | autotransporter adhesin Aae |
| D7S_00966 | 0.005124662 | 1.51 | permease |
| D7S_00896 | 6.63279E-05 | 1.51 | sigma-E factor negative regulatory protein |
| D7S_00236 | 0.000943555 | 1.50 | CitT protein |
| D7S_01937 | 0.001648783 | 1.50 | heme ABC superfamily ATP binding cassette |
| D7S_01617 | 1.10765E-05 | -1.50 | pyrimidine regulatory protein PyrR |
| D7S_00631 | 0.000510422 | -1.50 | holliday junction DNA helicase B |
| D7S_01318 | 0.001734714 | -1.51 | nitrate/nitrite sensor protein NarQ |
| D7S_02421 | 5.30566E-05 | -1.51 | secreted protein |
| D7S_00333 | 0.027724046 | -1.51 | 3-deoxy-D-manno-octulosonate |
| D7S_01431 | 1.90347E-05 | -1.51 | transaldolase |
| D7S_00240 | 0.007483863 | -1.51 | molybdate ABC transporter ATP-binding protein |
| D7S_01097 | 0.01655484 | -1.51 | competence protein A |
| D7S_02218 | 0.003762701 | -1.52 | molybdopterin synthase sulfurylase MoeB |
| D7S_02166 | 0.008361807 | -1.52 | cell filamentation protein Fic-like protein |
| D7S_01334 | 0.011162759 | -1.52 | hypothetical protein |
| D7S_00091 | 0.020983579 | -1.52 | deoxyguanosinetriphosphate |
| D7S_02416 | 7.69726E-06 | -1.52 | long-chain-fatty-acid--CoA ligase |
| D7S_00900 | 0.00019664 | -1.52 | prolyl-tRNA synthetase |
| D7S_00959 | 0.014208691 | -1.53 | XylB protein |
| D7S_02034 | 0.001442066 | -1.53 | exodeoxyribonuclease VII large subunit |
| D7S_02227 | 0.005224419 | -1.53 | multidrug efflux protein |
| D7S_01025 | 0.007626268 | -1.53 | Holliday junction resolvase-like protein |
| D7S_01119 | 0.00436353 | -1.53 | hypothetical protein |
| D7S_00814 | 0.000400147 | -1.53 | diadenosine tetraphosphatase |
| D7S_02108 | 0.012696294 | -1.53 | hypothetical protein |
| D7S_00400 | 2.14347E-05 | -1.54 | N-acetylglucosamine-6-phosphate deacetylase |
| D7S_01149 | 0.00074089 | -1.54 | TonB system transport protein ExbD |
| D7S_00819 | 0.000823379 | -1.54 | magnesium and cobalt transport protein CorA |
| D7S_00437 | 0.000626757 | -1.54 | membrane protein |
| D7S_00815 | 0.000658589 | -1.55 | virulence-associated protein D |
| D7S_01364 | 1.90834E-05 | -1.55 | aspartate-semialdehyde dehydrogenase |
| D7S_00666 | 5.53467E-06 | -1.55 | SsrA-binding protein |
| D7S_00996 | 0.01896623 | -1.55 | aldo/keto reductase |
| D7S_00105 | 0.021253998 | -1.56 | MmcQ protein |
| D7S_02198 | 0.005028131 | -1.56 | WcaA protein |
| D7S_02413 | 0.00012672 | -1.56 | putative M22 peptidase-like protein YeaZ |
| D7S_01747 | 0.005325916 | -1.56 | putative protein insertion permease FtsX |
| D7S_00179 | 1.93876E-05 | -1.56 | HTH-type transcriptional regulator MetR |
| D7S_01798 | 6.59349E-05 | -1.56 | PerM family permease |
| D7S_01034 | 8.92631E-06 | -1.57 | NADH:ubiquinone oxidoreductase subunit C |
| D7S_00967 | 0.007008926 | -1.57 | phosphoglycerate mutase |
| D7S_01316 | 4.55085E-05 | -1.57 | RNA polymerase factor sigma-32 |
| D7S_00828 | 0.00327254 | -1.57 | 23S rRNA pseudouridine synthase D |
| D7S_01221 | 4.64442E-05 | -1.57 | PspE protein |
| D7S_00495 | 0.031634915 | -1.58 | lipoprotein |
| D7S_01160 | 1.01362E-06 | -1.58 | lipoprotein VacJ |
| D7S_00099 | 5.68287E-05 | -1.58 | transposase, IS4 family protein |
| D7S_00231 | 0.002780615 | -1.58 | putative RNA 2'-O-ribose methyltransferase MtfA |
| D7S_02381 | 0.000536709 | -1.58 | replicative DNA helicase |
| D7S_02018 | 0.015050102 | -1.59 | methylated-DNA--protein-cysteine |
| D7S_01864 | 0.005943732 | -1.59 | keto-hydroxyglutarate-aldolase/keto-deoxy- |
| D7S_01139 | 0.008730603 | -1.59 | MerR family transcriptional regulator |
| D7S_02077 | 0.005943732 | -1.59 | chaperone protein DnaJ |
| D7S_00653 | 0.042550282 | -1.59 | Na+/H+ antiporter NhaC |
| D7S_00012 | 0.000314668 | -1.59 | dethiobiotin synthase |
| D7S_02355 | 0.002923322 | -1.59 | cardiolipin synthetase |
| D7S_01174 | 6.81124E-05 | -1.59 | peptidyl-prolyl cis-trans isomerase |
| D7S_00881 | 0.000244193 | -1.59 | pantothenate kinase |
| D7S_00899 | 0.009124526 | -1.59 | DMT superfamily drug/metabolite transporter |
| D7S_00072 | 9.1014E-07 | -1.60 | chromosome partition protein MukF |
| D7S_01585 | 0.045153305 | -1.60 | hypothetical protein |
| D7S_00527 | 9.40519E-07 | -1.60 | class I and II aminotransferase |
| D7S_01132 | 3.71993E-06 | -1.61 | dimethyladenosine transferase |
| D7S_00807 | 0.000584505 | -1.61 | ADP compounds hydrolase NudE |
| D7S_02207 | 1.46503E-05 | -1.62 | NagC protein |
| D7S_00646 | 0.006182834 | -1.62 | brp/Blh family beta-carotene |
| D7S_02427 | 0.000323912 | -1.62 | cold shock-induced palmitoleoyl transferase |
| D7S_02215 | 0.029714819 | -1.63 | cysteine/glutathione ABC transporter |
| D7S_01621 | 1.47814E-06 | -1.63 | hypothetical protein |
| D7S_00708 | 4.30459E-06 | -1.63 | Fe-S protein assembly chaperone HscA |
| D7S_01568 | 7.85666E-06 | -1.63 | lipoprotein B |
| D7S_00820 | 0.003717389 | -1.63 | integral membrane protein |
| D7S_00339 | 2.52098E-07 | -1.63 | selenide, water dikinase |
| D7S_02184 | 0.014977408 | -1.63 | formate dehydrogenase family accessory protein |
| D7S_00902 | 2.16138E-05 | -1.64 | selenocysteine-specific translation elongation |
| D7S_01550 | 0.024859388 | -1.64 | inner membrane protein YbhI |
| D7S_01530 | 0.000428598 | -1.64 | ATP-NAD kinase |
| D7S_00321 | 0.000175078 | -1.65 | membrane protein yfbV |
| D7S_00245 | 0.025747386 | -1.65 | hypothetical protein |
| D7S_01462 | 1.65629E-05 | -1.65 | acetyltransferase |
| D7S_01696 | 0.000100219 | -1.65 | toxin-antitoxin system, toxin component, RelE |
| D7S_00981 | 0.003136848 | -1.66 | putative tubulin binding protein |
| D7S_00140 | 2.1936E-05 | -1.66 | transporting ATPase |
| D7S_01371 | 4.13353E-08 | -1.66 | glycyl-tRNA synthetase subunit beta |
| D7S_02356 | 0.00028777 | -1.66 | membrane protein |
| D7S_01628 | 2.05103E-07 | -1.66 | 2,3-bisphosphoglycerate-dependent |
| D7S_01250 | 0.004127937 | -1.66 | deoxyribose-phosphate aldolase |
| D7S_00070 | 1.53136E-07 | -1.67 | 2_acylglycerophosphoethanolamine |
| D7S_01087 | 0.013092909 | -1.68 | phosphopantothenoylcysteine |
| D7S_02216 | 7.33698E-08 | -1.68 | thioredoxin-disulfide reductase |
| D7S_00317 | 0.001944127 | -1.68 | maltodextrin phosphorylase |
| D7S_01026 | 2.32208E-07 | -1.68 | phosphopyruvate hydratase |
| D7S_01124 | 2.74765E-05 | -1.69 | RlpB protein |
| D7S_01009 | 6.15505E-07 | -1.69 | SmpA protein |
| D7S_01545 | 0.035810189 | -1.69 | galactokinase |
| D7S_00008 | 1.11512E-07 | -1.70 | stringent starvation protein B |
| D7S_00729 | 0.037220158 | -1.70 | D-ribose transporter subunit RbsB |
| D7S_00196 | 0.00036065 | -1.70 | WcaG protein |
| D7S_00015 | 0.023202846 | -1.70 | ribose import ATP-binding protein RbsA |
| D7S_00543 | 3.86186E-06 | -1.70 | CRISPR-associated Csy1 family protein |
| D7S_01740 | 2.28646E-05 | -1.70 | 1,4-dihydroxy-2-naphthoate |
| D7S_00487 | 0.000183656 | -1.70 | adenylate cyclase |
| D7S_01962 | 0.000202006 | -1.71 | tRNA modification GTPase TrmE |
| D7S_00995 | 3.95679E-07 | -1.71 | carboxylesterase type B |
| D7S_01331 | 1.13312E-06 | -1.71 | shikimate kinase |
| D7S_01338 | 5.42733E-05 | -1.72 | short chain dehydrogenase |
| D7S_00248 | 9.34784E-05 | -1.72 | hypothetical protein |
| D7S_01920 | 1.24487E-05 | -1.72 | 2-succinyl-6-hydroxy-2, |
| D7S_00325 | 0.048182531 | -1.72 | putative rmlC-like jelly roll fold-containing |
| D7S_02160 | 0.039455293 | -1.72 | hypothetical protein |
| D7S_01575 | 9.30966E-06 | -1.72 | tryptophanyl-tRNA synthetase |
| D7S_01344 | 6.54236E-05 | -1.72 | thioesterase |
| D7S_01131 | 1.35377E-06 | -1.72 | diadenosine tetraphosphatase |
| D7S_01854 | 8.91702E-09 | -1.72 | isoleucyl-tRNA synthetase |
| D7S_00810 | 1.88269E-06 | -1.73 | oligopeptide transporter |
| D7S_01970 | 2.11616E-08 | -1.73 | 6-phosphofructokinase |
| D7S_00673 | 0.039842512 | -1.73 | oligopeptide transport ATP-binding protein OppD |
| D7S_01288 | 3.8588E-08 | -1.73 | PTS system mannose-specific EIIAB component |
| D7S_01980 | 5.37864E-07 | -1.73 | pyruvate formate-lyase activating enzyme |
| D7S_02274 | 0.002681125 | -1.73 | DNA mismatch repair protein MutL |
| D7S_00222 | 0.026725971 | -1.74 | electron transport complex protein RnfB |
| D7S_01606 | 1.98533E-07 | -1.74 | phosphatase YbhA |
| D7S_01860 | 0.000594913 | -1.74 | putative oxygen-independent coproporphyrinogen |
| D7S_01361 | 4.3997E-05 | -1.74 | lipoprotein |
| D7S_02105 | 0.005543476 | -1.74 | YccS/YhfK family integral membrane protein |
| D7S_01954 | 1.19534E-08 | -1.74 | copper/zinc superoxide dismutase |
| D7S_00114 | 0.000228974 | -1.74 | exodeoxyribonuclease V subunit alpha |
| D7S_00003 | 1.20108E-08 | -1.74 | molybdenum cofactor biosynthesis protein A |
| D7S_01351 | 2.05579E-08 | -1.75 | acyl carrier protein |
| D7S_00612 | 0.000124915 | -1.75 | DNA polymerase III subunit psi |
| D7S_01922 | 2.15656E-08 | -1.76 | 30S ribosomal protein S15 |
| D7S_00430 | 8.87771E-09 | -1.76 | hydroxyacylglutathione hydrolase |
| D7S_00397 | 3.77713E-06 | -1.77 | ferrochelatase |
| D7S_01631 | 0.001467394 | -1.77 | divergent polysaccharide deacetylase |
| D7S_01057 | 7.00809E-07 | -1.78 | K+-transporting ATPase |
| D7S_01695 | 0.014144464 | -1.78 | putative DNA-binding protein |
| D7S_01492 | 5.12373E-06 | -1.78 | bifunctional aspartokinase I/homoserine |
| D7S_01098 | 0.015586371 | -1.79 | ComB, putative |
| D7S_01858 | 0.000465482 | -1.79 | non-canonical purine NTP pyrophosphatase, |
| D7S_00448 | 9.59844E-06 | -1.79 | DNA topoisomerase IV subunit A |
| D7S_01861 | 9.52791E-08 | -1.79 | ribose 5-phosphate isomerase A |
| D7S_00842 | 7.39221E-10 | -1.80 | ribonuclease R |
| D7S_01226 | 0.036549219 | -1.80 | fructose-1,6-bisphosphatase |
| D7S_01649 | 2.90595E-05 | -1.81 | putative ATPase |
| D7S_01746 | 0.019037633 | -1.81 | hypothetical protein |
| D7S_01224 | 1.45976E-05 | -1.81 | serine acetyltransferase |
| D7S_01751 | 0.008943884 | -1.81 | putative methyltransferase |
| D7S_01983 | 1.45976E-05 | -1.82 | 1-acyl-sn-glycerol-3-phosphate acyltransferase |
| D7S_00808 | 0.008134437 | -1.82 | histidine kinase |
| D7S_02009 | 4.41878E-05 | -1.82 | glycine betaine |
| D7S_01072 | 6.28935E-07 | -1.82 | cell division protein FtsN |
| D7S_02264 | 0.008943884 | -1.82 | peptide transport system permease SapB |
| D7S_01827 | 0.040424993 | -1.82 | integrase |
| D7S_00079 | 0.04457879 | -1.83 | glucose-1-phosphate adenylyltransferase |
| D7S_00958 | 0.04457879 | -1.83 | monosaccharide-transporting ATPase |
| D7S_00470 | 2.8815E-05 | -1.83 | putative cryptic C4-dicarboxylate transporter |
| D7S_01943 | 5.22415E-07 | -1.84 | hypothetical protein |
| D7S_00330 | 0.00012423 | -1.84 | lipid A export permease/ATP-binding protein |
| D7S_01151 | 2.77809E-06 | -1.84 | HtpX protein |
| D7S_01572 | 0.003579201 | -1.84 | cell division protein FtsB |
| D7S_01386 | 1.61013E-07 | -1.84 | aspartate-semialdehyde dehydrogenase |
| D7S_00331 | 4.02244E-05 | -1.84 | tetraacyldisaccharide 4'-kinase |
| D7S_01136 | 0.000599999 | -1.84 | protein YcfC |
| D7S_01405 | 0.048952148 | -1.85 | integrase |
| D7S_00873 | 0.014053129 | -1.85 | acetohydroxy acid synthase II |
| D7S_00871 | 3.92085E-06 | -1.85 | RfaL protein |
| D7S_00090 | 0.000930695 | -1.85 | dGTP triphosphohydrolase |
| D7S_01095 | 7.37079E-10 | -1.85 | tyrosine recombinase XerD |
| D7S_01842 | 3.65434E-05 | -1.85 | uracil-DNA glycosyllase |
| D7S_01191 | 1.61164E-10 | -1.86 | ATP-dependent RNA helicase RhlB |
| D7S_01748 | 0.000534005 | -1.86 | cell division ATP-binding protein FtsE |
| D7S_00797 | 2.61991E-07 | -1.86 | twin arginine translocase protein A |
| D7S_00214 | 0.001879008 | -1.86 | high-affinity zinc uptake system membrane |
| D7S_00737 | 1.06605E-10 | -1.86 | argininosuccinate lyase |
| D7S_01499 | 2.57912E-05 | -1.87 | alcohol dehydrogenase |
| D7S_00813 | 6.66861E-12 | -1.87 | glucose-6-phosphate 1-dehydrogenase |
| D7S_01194 | 1.35921E-10 | -1.87 | penicillin-binding protein 2 |
| D7S_01460 | 1.71247E-08 | -1.88 | hypothetical protein |
| D7S_00768 | 0.007074062 | -1.88 | IMPACT family protein |
| D7S_00191 | 0.002076188 | -1.88 | glutamate-ammonia-ligase adenylyltransferase |
| D7S_01694 | 3.1576E-11 | -1.88 | ATP-dependent Clp protease ATP-binding subunit |
| D7S_02284 | 0.009640082 | -1.89 | integrase |
| D7S_00223 | 0.001694039 | -1.89 | Electron transport complex protein rnfA |
| D7S_00964 | 7.78812E-11 | -1.89 | integrase, catalytic region |
| D7S_00219 | 0.00219157 | -1.89 | electron transport complex protein RnfG |
| D7S_01263 | 0.00016052 | -1.89 | hypothetical protein |
| D7S_01569 | 3.8605E-11 | -1.89 | 5'/3'-nucleotidase SurE |
| D7S_00477 | 0.000195583 | -1.90 | DNA mismatch repair protein |
| D7S_01766 | 1.34427E-05 | -1.90 | regulatory protein UhpC |
| D7S_00544 | 0.014053129 | -1.90 | CRISPR-associated Csy2 family protein |
| D7S_01004 | 0.002354859 | -1.90 | ribonuclease BN |
| D7S_02107 | 9.41839E-06 | -1.90 | methylglyoxal synthase |
| D7S_00021 | 2.40028E-10 | -1.91 | pyruvate kinase |
| D7S_00532 | 2.10584E-07 | -1.92 | protein-export membrane protein SecF |
| D7S_00073 | 0.014053129 | -1.92 | DacC protein |
| D7S_01836 | 0.000358263 | -1.92 | NAD-dependent DNA ligase |
| D7S_00994 | 0.002354859 | -1.92 | putative exported protein precursor |
| D7S_01395 | 5.31224E-07 | -1.92 | S-ribosylhomocysteinase |
| D7S_00109 | 0.006895724 | -1.92 | putative plasmid stability protein StbE, |
| D7S_01192 | 5.38665E-08 | -1.93 | iojap-like protein |
| D7S_01030 | 1.0963E-11 | -1.93 | thiamine biosynthesis lipoprotein ApbE |
| D7S_01923 | 8.5964E-05 | -1.93 | methionine adenosyltransferase |
| D7S_01443 | 8.48436E-09 | -1.93 | transposase |
| D7S_01582 | 0.043313515 | -1.94 | hypothetical protein |
| D7S_00548 | 0.007698293 | -1.94 | CRISPR-associated Cas1 family protein |
| D7S_00115 | 2.97476E-05 | -1.94 | exodeoxyribonuclease V subunit beta |
| D7S_00586 | 1.30296E-06 | -1.94 | orotidine 5'-phosphate decarboxylase / humps |
| D7S_01573 | 1.84221E-10 | -1.94 | ribulose-phosphate 3-epimerase |
| D7S_02404 | 0.022380974 | -1.95 | maltose transport system permease MalF |
| D7S_02090 | 0.013593404 | -1.95 | penicillin-binding protein 1C |
| D7S_01143 | 6.015E-07 | -1.95 | signal recognition particle protein |
| D7S_00607 | 6.52272E-10 | -1.95 | serine hydroxymethyltransferase |
| D7S_00005 | 9.14037E-08 | -1.95 | molybdopterin converting factor subunit 1 |
| D7S_01010 | 2.03713E-09 | -1.95 | nucleoid-associated protein NdpA |
| D7S_01675 | 7.52506E-07 | -1.96 | hypothetical protein |
| D7S_01692 | 0.006379941 | -1.96 | tRNA modification GTPase TrmE |
| D7S_01838 | 4.7844E-05 | -1.96 | cell division protein ZipA |
| D7S_01159 | 6.42287E-05 | -1.96 | nickel ABC transporter periplasmic |
| D7S_01339 | 3.35841E-05 | -1.96 | thioester dehydrase family protein |
| D7S_01605 | 2.016E-07 | -1.96 | DNA mismatch repair protein MutS |
| D7S_00569 | 0.000434024 | -1.97 | hypothetical protein |
| D7S_01016 | 2.70387E-08 | -1.97 | universal stress protein A |
| D7S_00917 | 0.001210147 | -1.97 | phosphatidate cytidylyltransferase |
| D7S_01459 | 1.4181E-10 | -1.98 | putative metalloprotease in flp 5' region |
| D7S_00506 | 0.000116329 | -1.98 | ribonuclease HI |
| D7S_00854 | 2.55796E-13 | -1.98 | glycerol-3-phosphate O-acyltransferase |
| D7S_01384 | 2.59782E-09 | -1.99 | DNA polymerase I |
| D7S_02199 | 2.45021E-09 | -1.99 | amylovoran biosynthesis glycosylltransferase |
| D7S_02102 | 3.97198E-07 | -1.99 | transketolase, central region |
| D7S_01463 | 8.59253E-09 | -1.99 | hypothetical protein |
| D7S_00883 | 3.77356E-07 | -1.99 | ATP-dependent DNA helicase RecQ |
| D7S_02269 | 0.000142415 | -1.99 | hypothetical protein |
| D7S_00589 | 2.09182E-05 | -1.99 | Rhs element Vgr protein |
| D7S_00006 | 8.42751E-09 | -2.00 | molybdopterin converting factor subunit 2 |
| D7S_00376 | 7.22219E-05 | -2.00 | hypothetical protein |
| D7S_01724 | 0.000621232 | -2.00 | protein AmpD |
| D7S_00546 | 7.806E-10 | -2.02 | Csy4 family CRISPR-associated protein |
| D7S_01979 | 6.33582E-12 | -2.02 | formate acetyltransferase |
| D7S_01557 | 7.03592E-11 | -2.02 | magnesium and cobalt efflux protein CorC |
| D7S_00493 | 0.005644555 | -2.02 | 4-diphosphocytidyl-2-C-methyl-D-erythritol |
| D7S_01430 | 2.56227E-13 | -2.02 | transketolase |
| D7S_02410 | 0.001428769 | -2.02 | aldose 1-epimerase |
| D7S_00207 | 1.65804E-11 | -2.03 | 2',3'-cyclic-nucleotide 2'-phosphodiesterase |
| D7S_02145 | 1.88139E-09 | -2.03 | spermidine/putrescine ABC transporter membrane |
| D7S_00445 | 5.97507E-13 | -2.03 | ABC transporter domain-containing protein |
| D7S_01326 | 0.00468195 | -2.03 | cytochrome c-type protein TorC |
| D7S_00914 | 4.19304E-06 | -2.03 | hypothetical protein |
| D7S_00156 | 1.08656E-06 | -2.05 | inner membrane protein YfcA |
| D7S_01126 | 1.63173E-13 | -2.05 | leucyl-tRNA synthetase |
| D7S_02350 | 1.42046E-07 | -2.06 | geranyltranstransferase |
| D7S_01445 | 8.55416E-10 | -2.06 | Flp pilus assembly protein TadF |
| D7S_00224 | 0.00721512 | -2.07 | NAD-dependent epimerase/dehydratase |
| D7S_01486 | 7.02749E-12 | -2.07 | cell division protein FtsZ |
| D7S_02339 | 3.91198E-08 | -2.07 | ferredoxin--NADP reductase |
| D7S_01958 | 5.7313E-13 | -2.07 | chromosomal replication initiator protein DnaA |
| D7S_01155 | 0.004957611 | -2.08 | hypothetical protein |
| D7S_02162 | 1.97632E-06 | -2.09 | succinyl-CoA ligase |
| D7S_01769 | 0.049970825 | -2.09 | transposase, IS4 family protein |
| D7S_01337 | 2.79779E-06 | -2.10 | beta-ketoacyl-ACP synthase IV |
| D7S_01102 | 1.79408E-12 | -2.10 | shikimate kinase |
| D7S_00678 | 0.000303747 | -2.10 | decarboxylase |
| D7S_01000 | 6.52662E-05 | -2.11 | ATP-binding protein |
| D7S_01726 | 0.002147733 | -2.11 | EbgC protein |
| D7S_02209 | 3.14673E-10 | -2.11 | outer-membrane lipoprotein carrier protein |
| D7S_01199 | 5.04807E-12 | -2.11 | lipoyltransferase |
| D7S_00247 | 9.82859E-05 | -2.12 | hypothetical protein |
| D7S_01567 | 2.9667E-10 | -2.12 | outer membrane antigenic lipoprotein B |
| D7S_02066 | 1.94355E-12 | -2.12 | ribonuclease, Rne/Rng family |
| D7S_00531 | 3.98225E-16 | -2.12 | protein-export membrane protein SecD |
| D7S_00346 | 1.57224E-08 | -2.12 | tyrosyl-tRNA synthetase |
| D7S_02384 | 0.003730577 | -2.13 | hypothetical protein |
| D7S_02093 | 0.003011952 | -2.13 | Rhs element Vgr protein |
| D7S_01956 | 1.80569E-10 | -2.13 | DNA replication and repair protein RecF |
| D7S_02134 | 4.8647E-06 | -2.13 | thiamine ABC transporter ATP-binding protein |
| D7S_02275 | 7.65038E-07 | -2.14 | N-acetylmuramoyl-L-alanine amidase |
| D7S_02060 | 2.5533E-09 | -2.14 | GTP-binding protein EngA |
| D7S_01654 | 1.33505E-07 | -2.15 | ribosomal large subunit pseudouridine synthase |
| D7S_01426 | 5.47302E-05 | -2.15 | ABC transporter ATP-binding protein |
| D7S_01497 | 0.000640253 | -2.15 | ExsB protein |
| D7S_00903 | 2.75656E-14 | -2.16 | lipoprotein |
| D7S_02062 | 3.31557E-07 | -2.16 | bacterial regulatory protein, MerR family |
| D7S_01577 | 0.027651377 | -2.16 | sodium-dependent transporter |
| D7S_00712 | 0.002580514 | -2.16 | Mrp ATPase family protein |
| D7S_01104 | 2.83632E-05 | -2.16 | DNA adenine methylase |
| D7S_00218 | 6.5876E-07 | -2.16 | electron transport complex protein RnfE |
| D7S_02114 | 1.35947E-09 | -2.17 | sulfurtransferase TusE |
| D7S_02311 | 5.60176E-05 | -2.17 | oligoribonuclease |
| D7S_02178 | 3.41223E-05 | -2.17 | HypF protein |
| D7S_00947 | 1.68332E-05 | -2.17 | RarD protein |
| D7S_00946 | 0.000535289 | -2.17 | protein YrdC |
| D7S_01895 | 1.20775E-08 | -2.18 | serine transporter |
| D7S_00498 | 2.12484E-10 | -2.18 | hypothetical protein |
| D7S_00494 | 3.14027E-09 | -2.18 | ribose-Phosphate pyrophosphokinase |
| D7S_00299 | 7.52745E-09 | -2.18 | HTH-type transcriptional repressor PurR |
| D7S_01329 | 1.57666E-06 | -2.18 | filamentation induced by cAMP protein fic |
| D7S_01720 | 0.004689174 | -2.19 | prepilin peptidase |
| D7S_00919 | 6.5876E-07 | -2.19 | CDP-alcohol phosphatidyltransferase |
| D7S_02002 | 1.20952E-05 | -2.19 | hypothetical protein |
| D7S_01650 | 1.071E-07 | -2.20 | ATP-dependent RNA helicase SrmB |
| D7S_02059 | 0.001571867 | -2.21 | sugar efflux transporter B |
| D7S_01330 | 4.70678E-12 | -2.21 | ADP-glyceromanno-heptose 6-epimerase |
| D7S_02266 | 1.87066E-06 | -2.21 | peptide ABC superfamily ATP binding cassette |
| D7S_00754 | 1.42083E-15 | -2.21 | cysteinyl-tRNA synthetase |
| D7S_00833 | 3.4817E-10 | -2.22 | yecA family protein |
| D7S_01630 | 4.87393E-07 | -2.22 | NlpD protein |
| D7S_00004 | 1.62907E-10 | -2.23 | molybdenum cofactor biosynthesis protein C |
| D7S_01158 | 1.12123E-15 | -2.23 | ATP-dependent protease ATP-binding subunit HslU |
| D7S_02000 | 0.008028763 | -2.23 | putative 4'-phosphopantetheinyl transferase |
| D7S_00036 | 2.69673E-07 | -2.23 | plasmid maintenance system antidote protein |
| D7S_00599 | 0.000324003 | -2.25 | mannitol repressor protein |
| D7S_01538 | 0.016669352 | -2.25 | galactoside transport system permease protein |
| D7S_00132 | 0.016669352 | -2.26 | ISPsy8, transposase OrfA |
| D7S_00435 | 4.81452E-05 | -2.26 | mannose-6-phosphate isomerase |
| D7S_00486 | 2.64774E-06 | -2.27 | DNA repair protein RadA |
| D7S_01940 | 3.41942E-05 | -2.27 | cytochrome c-type biogenesis protein CcmF |
| D7S_00979 | 1.01894E-12 | -2.27 | nitrate/nitrite response regulator protein NarP |
| D7S_01919 | 3.78266E-09 | -2.29 | menaquinone-specific isochorismate synthase |
| D7S_01603 | 0.001325303 | -2.29 | hypothetical protein |
| D7S_00945 | 8.09081E-05 | -2.29 | DNA topoisomerase I |
| D7S_01169 | 1.14668E-11 | -2.29 | dihydrofolate reductase |
| D7S_01972 | 5.91217E-14 | -2.30 | 6-phosphofructokinase |
| D7S_00071 | 1.52401E-06 | -2.31 | hypothetical protein |
| D7S_01975 | 1.91143E-09 | -2.31 | beta-hexosaminidase |
| D7S_00869 | 1.4466E-19 | -2.32 | phosphoglycerate kinase |
| D7S_01219 | 1.3551E-07 | -2.32 | hypothetical protein |
| D7S_01286 | 3.39304E-18 | -2.33 | mannose-specific PTS system protein IID |
| D7S_01974 | 1.59298E-09 | -2.34 | 23S rRNA methyluridine methyltransferase |
| D7S_01885 | 0.00054412 | -2.35 | isoprenylcysteine carboxyl methyltransferase |
| D7S_02393 | 4.22418E-17 | -2.35 | ribonucleoside-diphosphate reductase subunit |
| D7S_01362 | 5.10262E-14 | -2.35 | ATP-dependent chaperone ClpB |
| D7S_01625 | 0.000103657 | -2.36 | Sel1 domain-containing protein repeat-containing |
| D7S_01062 | 5.6262E-05 | -2.36 | DNA repair protein RecO low-quality sequence region |
| D7S_02230 | 0.011385303 | -2.36 | tRNA pseudouridine synthase C |
| D7S_01566 | 1.17905E-18 | -2.36 | outer membrane antigenic lipoprotein B |
| D7S_00379 | 1.02438E-17 | -2.36 | hypothetical protein |
| D7S_00634 | 3.18022E-13 | -2.37 | Holliday junction resolvase |
| D7S_00898 | 2.08111E-07 | -2.39 | TPR repeat protein |
| D7S_00483 | 1.4108E-05 | -2.39 | AmpG protein |
| D7S_00572 | 0.007964202 | -2.39 | GrxA family glutaredoxin |
| D7S_01255 | 1.31051E-12 | -2.39 | paar repeat-containing protein |
| D7S_00205 | 0.022271588 | -2.40 | hypothetical protein |
| D7S_02017 | 8.27572E-08 | -2.40 | DNA mismatch repair protein |
| D7S_00203 | 6.00856E-08 | -2.40 | transcriptional regulatory protein QseB |
| D7S_02263 | 7.26796E-11 | -2.40 | dipeptide transport system permease DppC |
| D7S_01505 | 1.47585E-19 | -2.41 | lysyl-tRNA synthetase |
| D7S_00386 | 0.029036359 | -2.41 | putative phosphatase |
| D7S_00013 | 2.83358E-09 | -2.42 | L-ribulokinase |
| D7S_02101 | 0.000179955 | -2.42 | transketolase |
| D7S_00517 | 2.81186E-05 | -2.42 | hypothetical protein |
| D7S_00504 | 0.033655011 | -2.42 | mobilization protein A |
| D7S_02103 | 5.55296E-09 | -2.43 | N5-glutamine S-adenosyl-L-methionine-dependent |
| D7S_01750 | 2.36547E-18 | -2.44 | cell division protein FtsY |
| D7S_01076 | 2.31595E-09 | -2.44 | lipooligosaccharide biosynthesis protein lex-1 |
| D7S_01287 | 5.10538E-20 | -2.45 | PTS system mannose/fructose/sorbose family |
| D7S_00092 | 1.18788E-12 | -2.46 | membrane protein |
| D7S_01802 | 8.35946E-07 | -2.46 | tRNA-dihydrouridine synthase A |
| D7S_00855 | 7.19089E-20 | -2.48 | MdlB protein |
| D7S_01950 | 0.002625922 | -2.48 | cytochrome c-type biogenesis protein CcmF |
| D7S_01655 | 2.8554E-19 | -2.49 | Rhs element Vgr protein |
| D7S_00227 | 5.18444E-16 | -2.51 | malate dehydrogenase |
| D7S_02055 | 2.81196E-09 | -2.52 | cystathionine beta-lyase |
| D7S_01574 | 1.1071E-17 | -2.52 | phosphoglycolate phosphatase |
| D7S_02058 | 1.33532E-06 | -2.52 | sugar efflux transporter |
| D7S_00259 | 2.16011E-19 | -2.53 | thiol-disulfide interchange protein |
| D7S_00570 | 3.04762E-17 | -2.53 | ribosomal protein S6 modification protein |
| D7S_01396 | 2.05176E-13 | -2.54 | hypothetical protein |
| D7S_01137 | 0.000592405 | -2.54 | adenylosuccinate lyase |
| D7S_02332 | 9.78048E-08 | -2.54 | fructosamine kinase |
| D7S_00221 | 6.14715E-06 | -2.54 | electron transport complex protein RnfC |
| D7S_01982 | 4.8263E-08 | -2.55 | protein SufI |
| D7S_01113 | 8.25177E-21 | -2.55 | excinuclease ABC subunit A |
| D7S_00926 | 5.68873E-19 | -2.55 | outer membrane protein 26 |
| D7S_00246 | 2.25369E-12 | -2.55 | shikimate 5-dehydrogenase |
| D7S_01365 | 8.40698E-05 | -2.56 | Cof protein |
| D7S_01163 | 9.68844E-06 | -2.56 | Rhs element Vgr protein |
| D7S_02405 | 1.052E-12 | -2.58 | maltose ABC transporter periplasmic protein |
| D7S_02217 | 1.77719E-09 | -2.58 | thioredoxin domain-containing protein |
| D7S_00049 | 2.11528E-06 | -2.58 | putative FHA domain protein |
| D7S_01088 | 0.004693354 | -2.59 | hypothetical protein |
| D7S_00431 | 8.04462E-14 | -2.60 | SmtA protein |
| D7S_00020 | 2.38007E-20 | -2.60 | autoinducer-2 modifying protein LsrG |
| D7S_02396 | 1.89929E-12 | -2.61 | ribonucleoside-diphosphate reductase subunit |
| D7S_02144 | 3.52793E-19 | -2.62 | spermidine/putrescine-binding periplasmic |
| D7S_01289 | 5.66976E-06 | -2.62 | inner membrane transport protein YieO |
| D7S_02330 | 5.23151E-11 | -2.63 | glycerate dehydrogenase |
| D7S_00831 | 1.30584E-05 | -2.63 | 5-formyltetrahydrofolate cyclo-ligase |
| D7S_01495 | 8.34723E-06 | -2.64 | hypothetical protein |
| D7S_01909 | 0.000592405 | -2.64 | zinc protease |
| D7S_00081 | 2.33767E-11 | -2.65 | 1_4_alpha-glucan-branching protein |
| D7S_00916 | 0.001416628 | -2.65 | molybdopterin biosynthesis mog protein |
| D7S_02111 | 7.7212E-05 | -2.66 | putative ABC-type chelated iron transport |
| D7S_00574 | 7.48526E-10 | -2.66 | tyrosine-specific transporter |
| D7S_00829 | 3.56436E-11 | -2.67 | ribosomal large subunit pseudouridine synthase |
| D7S_00840 | 4.41985E-10 | -2.67 | rod shape-determining protein MreC |
| D7S_02408 | 0.000811764 | -2.69 | maltose operon periplasmic protein |
| D7S_00216 | 7.87062E-08 | -2.69 | endonuclease III |
| D7S_00728 | 0.016183483 | -2.70 | ribose ABC transporter permease |
| D7S_01246 | 6.09635E-08 | -2.72 | anhydro-N-acetylmuramic acid kinase |
| D7S_02001 | 2.98345E-06 | -2.73 | threonine synthase |
| D7S_01508 | 2.00536E-05 | -2.73 | iron-dicitrate transporter subunit FecD |
| D7S_00233 | 1.09499E-14 | -2.73 | riboflavin biosynthesis protein RibD |
| D7S_01843 | 3.14914E-06 | -2.73 | hypothetical protein |
| D7S_00608 | 5.10216E-12 | -2.74 | lipoprotein |
| D7S_00891 | 1.54207E-22 | -2.75 | aminoacyl-histidine dipeptidase |
| D7S_00528 | 0.016102155 | -2.76 | hypothetical protein |
| D7S_01532 | 5.72015E-25 | -2.76 | co-chaperone GrpE |
| D7S_00738 | 4.67894E-08 | -2.78 | glutamate dehydrogenase |
| D7S_00568 | 2.52448E-16 | -2.80 | dihydroorotate dehydrogenase 2 |
| D7S_00188 | 0.001929743 | -2.81 | CRISPR-associated Cas5 family protein |
| D7S_00016 | 0.01467859 | -2.81 | inner membrane ABC transporter permease protein |
| D7S_00870 | 3.57436E-26 | -2.83 | fructose-bisphosphate aldolase |
| D7S_00475 | 7.48048E-26 | -2.84 | acetyl-CoA carboxylase, carboxyl transferase |
| D7S_00795 | 0.000156321 | -2.84 | twin arginine-targeting protein translocase |
| D7S_02063 | 5.27101E-28 | -2.85 | adenylosuccinate synthetase |
| D7S_00794 | 1.5126E-19 | -2.86 | delta-aminolevulinic acid dehydratase |
| D7S_01228 | 2.80422E-09 | -2.86 | protein MioC |
| D7S_01461 | 4.27752E-24 | -2.87 | lipoprotein |
| D7S_00206 | 8.84106E-06 | -2.87 | formate dehydrogenase accessory protein FdhE |
| D7S_00382 | 0.003956865 | -2.88 | integral membrane protein-permease component, |
| D7S_01971 | 1.68024E-23 | -2.88 | ferrous-iron efflux pump FieF |
| D7S_00250 | 2.43958E-19 | -2.88 | hydrogenase expression/formation protein HypD |
| D7S_02057 | 1.71736E-25 | -2.88 | deoxycytidine triphosphate deaminase |
| D7S_00727 | 0.000541346 | -2.89 | D-ribose transporter ATP-binding protein |
| D7S_02061 | 8.15387E-10 | -2.93 | curved DNA-binding protein |
| D7S_01917 | 1.17447E-27 | -2.93 | fatty acid metabolism transcriptional regulator |
| D7S_01624 | 2.05958E-11 | -2.93 | Sel1 domain-containing protein repeat-containing |
| D7S_00019 | 5.15631E-08 | -2.93 | aldolase |
| D7S_01741 | 2.99823E-05 | -2.95 | protein YaeB |
| D7S_00018 | 2.82329E-06 | -2.95 | rhamnose ABC transporter, rhamnose-binding |
| D7S_02403 | 2.23248E-06 | -2.96 | maltose transport system permease MalG |
| D7S_01571 | 1.46442E-06 | -2.96 | 2-C-methyl-D-erythritol 4-phosphate |
| D7S_01735 | 1.43523E-14 | -2.97 | hypoxanthine-guanine phosphoribosyltransferase |
| D7S_00857 | 0.002952656 | -2.97 | alkylhydroperoxidase AhpD core |
| D7S_01340 | 2.80695E-11 | -2.97 | beta-ketoacyl synthase |
| D7S_01659 | 4.33201E-28 | -2.98 | WecD protein |
| D7S_01799 | 5.31841E-25 | -2.99 | uridine phosphorylase |
| D7S_00447 | 1.3925E-23 | -2.99 | DNA topoisomerase IV subunit B |
| D7S_00332 | 2.48346E-06 | -2.99 | tetraacyldisaccharide 4'-kinase |
| D7S_01894 | 9.61807E-15 | -3.02 | L-serine ammonia-lyase |
| D7S_01833 | 2.49366E-12 | -3.04 | O-sialoglycoprotein endopeptidase |
| D7S_01170 | 6.71295E-10 | -3.04 | YjjB like protein |
| D7S_00055 | 2.00435E-13 | -3.05 | aspartate aminotransferase |
| D7S_00585 | 1.14118E-09 | -3.06 | ascorbate-specific phosphotransferase enzyme iia |
| D7S_00841 | 3.60881E-20 | -3.08 | rod shape-determining protein MreB |
| D7S_01862 | 1.94682E-29 | -3.08 | D-3-phosphoglycerate dehydrogenase |
| D7S_02402 | 4.85605E-06 | -3.10 | periplasmic alpha-amylase |
| D7S_01772 | 0.031833594 | -3.10 | transposase |
| D7S_00185 | 1.78074E-05 | -3.14 | CRISPR-associated protein Cas4 |
| D7S_00547 | 1.31422E-06 | -3.14 | helicase |
| D7S_02004 | 9.64825E-30 | -3.18 | bifunctional aspartokinase I/homoserine |
| D7S_01947 | 4.42266E-13 | -3.22 | cytochrome c nitrite reductase, pentaheme |
| D7S_02056 | 3.10891E-30 | -3.24 | uridine kinase |
| D7S_02125 | 2.87999E-12 | -3.26 | CRISPR-associated protein Cas5 |
| D7S_01834 | 7.84212E-14 | -3.27 | hypothetical protein |
| D7S_00811 | 1.1308E-27 | -3.29 | hypothetical protein |
| D7S_00201 | 0.002114952 | -3.32 | sensor protein QseC |
| D7S_00131 | 0.008691218 | -3.36 | ISPsy8, transposase OrfA |
| D7S_01787 | 2.72192E-29 | -3.38 | methionyl-tRNA formyltransferase |
| D7S_02067 | 5.08192E-12 | -3.38 | GrxB family glutaredoxin |
| D7S_00336 | 0.000296958 | -3.40 | PotC protein |
| D7S_00324 | 1.89078E-22 | -3.40 | phosphate acetyltransferase |
| D7S_00567 | 5.26028E-14 | -3.41 | class II fumarate hydratase |
| D7S_00948 | 1.69605E-12 | -3.45 | shikimate 5-dehydrogenase |
| D7S_00749 | 2.22785E-14 | -3.46 | anaerobic ribonucleotide reductase-activating |
| D7S_01489 | 9.05811E-22 | -3.47 | heat shock protein 90 |
| D7S_00744 | 9.32963E-15 | -3.60 | putative long-chain-fatty-acid--CoA ligase-like |
| D7S_01906 | 3.29777E-20 | -3.65 | malic enzyme |
| D7S_00378 | 1.11069E-13 | -3.65 | high-affinity Fe2+/Pb2+ permease |
| D7S_00380 | 0.000113521 | -3.65 | integral membrane protein |
| D7S_00387 | 1.20915E-14 | -3.66 | hypothetical protein |
| D7S_02106 | 2.92878E-22 | -3.68 | inner membrane protein YccF |
| D7S_01380 | 1.14321E-06 | -3.68 | anaerobic dimethyl sulfoxide reductase chain C |
| D7S_01976 | 8.5598E-23 | -3.68 | lipoprotein |
| D7S_00384 | 1.50021E-05 | -3.70 | putative thioredoxin |
| D7S_01698 | 9.19896E-20 | -3.70 | copper-translocating P-type ATPase |
| D7S_00671 | 0.00409401 | -3.70 | oligopeptide transport system permease OppB |
| D7S_00017 | 0.002505547 | -3.72 | ABC transporter, permease, sugar transport |
| D7S_01697 | 7.13343E-14 | -3.72 | hypothetical protein |
| D7S_01058 | 1.92146E-22 | -3.75 | D-3-phosphoglycerate dehydrogenase |
| D7S_00186 | 0.00409401 | -3.76 | CRISPR-associated Csd2 family protein |
| D7S_01527 | 7.84725E-23 | -3.78 | outer membrane protein W |
| D7S_00610 | 0.012788659 | -3.78 | PTS system sucrose-specific transporter subunit |
| D7S_01531 | 6.6561E-13 | -3.85 | hypothetical protein |
| D7S_00500 | 1.59194E-40 | -3.92 | phosphoserine transaminase |
| D7S_02068 | 5.85814E-16 | -3.93 | Sir2 family transcriptional regulator |
| D7S_01911 | 6.5703E-15 | -3.94 | putative glycerol-3-phosphate acyltransferase |
| D7S_02164 | 6.14167E-35 | -3.98 | formate dehydrogenase subunit alpha |
| D7S_00383 | 2.42522E-08 | -4.01 | macrolide export ATP-binding/permease protein |
| D7S_02165 | 6.26421E-38 | -4.04 | formate dehydrogenase H |
| D7S_01618 | 7.96441E-45 | -4.06 | thymidylate kinase |
| D7S_01957 | 1.91241E-45 | -4.09 | DNA polymerase III subunit beta |
| D7S_01327 | 2.85676E-18 | -4.12 | bifunctional glutathionylspermidine |
| D7S_00252 | 5.94125E-42 | -4.19 | cobalt transport ATP-binding protein CbiO |
| D7S_01230 | 5.43441E-28 | -4.19 | hypothetical protein |
| D7S_01515 | 7.70577E-07 | -4.21 | hypothetical protein |
| D7S_00606 | 1.06564E-29 | -4.22 | serine hydroxymethyltransferase |
| D7S_02109 | 4.1323E-28 | -4.22 | acylphosphatase |
| D7S_00501 | 2.1229E-41 | -4.30 | histidinol-phosphate aminotransferase |
| D7S_00901 | 3.69194E-18 | -4.37 | L-seryl-tRNA selenium transferase |
| D7S_01805 | 3.00651E-29 | -4.38 | carbamate kinase |
| D7S_00014 | 5.7635E-42 | -4.39 | DeoR family transcriptional regulator |
| D7S_01949 | 1.09169E-17 | -4.39 | NrfD protein |
| D7S_02163 | 7.30682E-41 | -4.40 | hypothetical protein |
| D7S_02129 | 3.00973E-20 | -4.41 | CRISPR-associated protein Cas4 |
| D7S_01576 | 5.89039E-50 | -4.45 | 3,4-dihydroxy-2-butanone 4-phosphate synthase |
| D7S_00512 | 4.02829E-10 | -4.46 | cupin |
| D7S_02141 | 2.39672E-18 | -4.48 | C4-dicarboxylate membrane transporter |
| D7S_01029 | 2.62554E-43 | -4.52 | hypothetical protein |
| D7S_01341 | 2.04964E-14 | -4.57 | lipoprotein |
| D7S_01220 | 4.74407E-32 | -4.59 | anaerobic C4-dicarboxylate membrane transporter |
| D7S_02418 | 2.05725E-39 | -4.62 | cytochrome c peroxidase |
| D7S_00999 | 3.89661E-05 | -4.65 | O-methyltransferase domain-containing protein |
| D7S_01528 | 2.62797E-26 | -4.75 | outer membrane protein W |
| D7S_01629 | 4.89299E-49 | -4.78 | phosphoglucomutase/phosphomannomutase |
| D7S_02127 | 3.56554E-19 | -4.89 | crispr-associated protein, Csd1 family |
| D7S_00978 | 4.20613E-44 | -4.90 | adenylate cyclase, class I |
| D7S_01578 | 5.4735E-22 | -4.92 | RNA pseudouridine synthase family protein |
| D7S_01801 | 2.55835E-22 | -4.93 | asparagine synthetase AsnA |
| D7S_01535 | 7.46987E-11 | -4.96 | 5-methylaminomethyl-2-thiouridine |
| D7S_01103 | 7.42956E-51 | -4.96 | 3-dehydroquinate synthase |
| D7S_01681 | 8.02044E-32 | -5.03 | hypothetical protein |
| D7S_01841 | 1.27916E-07 | -5.09 | hypothetical protein |
| D7S_00381 | 1.24249E-07 | -5.11 | membrane protein |
| D7S_01027 | 6.24224E-14 | -5.13 | hypothetical protein |
| D7S_02148 | 3.25683E-24 | -5.14 | peptidase T |
| D7S_01536 | 5.07258E-45 | -5.22 | beta-ketoacyl-acyl-carrier-protein synthase II |
| D7S_00050 | 2.34476E-19 | -5.27 | TolC protein |
| D7S_01425 | 2.79281E-21 | -5.30 | MarC family integral membrane protein |
| D7S_00505 | 6.66363E-20 | -5.44 | 3-phosphoshikimate 1-carboxyvinyltransferase |
| D7S_01946 | 1.57761E-20 | -5.47 | cytochrome c nitrite reductase |
| D7S_00668 | 1.09558E-59 | -5.58 | oligopeptide ABC transporter periplasmic |
| D7S_00031 | 5.05141E-20 | -5.59 | oxaloacetate decarboxylase gamma chain 3 |
| D7S_00106 | 4.51781E-07 | -5.61 | CitT protein |
| D7S_01245 | 5.87397E-08 | -5.63 | N-acetylmuramic acid 6-phosphate etherase |
| D7S_01381 | 8.26432E-21 | -5.73 | anaerobic dimethyl sulfoxide reductase subunit |
| D7S_00077 | 4.93966E-63 | -6.02 | glycogen synthase |
| D7S_00238 | 0.02150752 | -6.04 | molybdate ABC transporter periplasmic |
| D7S_01379 | 9.7498E-18 | -6.14 | Twin-arginine leader-binding protein DmsD |
| D7S_01892 | 0.000256148 | -6.19 | hypothetical protein |
| D7S_01699 | 3.79774E-44 | -6.19 | heavy metal-binding protein, putative |
| D7S_00204 | 1.06348E-08 | -6.26 | protein YgiW |
| D7S_01803 | 7.82766E-20 | -6.56 | EriC protein |
| D7S_00657 | 2.18738E-26 | -6.57 | 2-octaprenyl-3-methyl-6-methoxy-1,4-benzoquinol |
| D7S_02128 | 3.17881E-08 | -6.64 | crispr-associated protein, Csd2 family |
| D7S_01679 | 6.13026E-72 | -7.03 | sigma 54 modulation protein/ribosomal protein |
| D7S_00726 | 3.06085E-26 | -7.41 | D-ribose pyranase |
| D7S_01804 | 2.61972E-78 | -7.44 | ornithine carbamoyltransferase |
| D7S_01488 | 4.21671E-92 | -7.48 | P-protein |
| D7S_00053 | 3.7919E-59 | -8.14 | AcrA protein |
| D7S_00251 | 5.54444E-34 | -8.31 | hydrogenase nickel incorporation protein HypB |
| D7S_00748 | 3.2013E-110 | -8.45 | anaerobic ribonucleoside-triphosphate reductase |
| D7S_00103 | 1.73583E-44 | -8.51 | cytochrome c-type protein TorY |
| D7S_00255 | 6.019E-106 | -8.91 | 50S ribosomal protein L25 |
| D7S_01378 | 9.27258E-17 | -9.04 | ferredoxin |
| D7S_01504 | 5.8163E-89 | -9.12 | fumarate reductase flavoprotein subunit |
| D7S_00051 | 7.04987E-23 | -9.14 | ABC transporter |
| D7S_02169 | 4.99126E-97 | -9.38 | hydrogenase-4 subunit I |
| D7S_00199 | 2.91257E-71 | -9.41 | gamma-glutamyltransferase |
| D7S_02170 | 7.01971E-91 | -9.65 | hydrogenase-4 component H |
| D7S_01948 | 1.3033E-45 | -9.74 | cytochrome c nitrite reductase, Fe-S protein |
| D7S_00254 | 1.8898E-101 | -9.83 | hypothetical protein |
| D7S_00052 | 3.03236E-42 | -9.84 | CcmA protein |
| D7S_01503 | 4.8427E-111 | -10.92 | fumarate reductase iron-sulfur subunit |
| D7S_01908 | 5.9798E-133 | -11.07 | putative lipoprotein |
| D7S_02171 | 3.03442E-74 | -11.18 | hydrogenase-4 subunit G |
| D7S_00029 | 7.41934E-62 | -11.29 | oxaloacetate decarboxylase subunit beta |
| D7S_02173 | 5.2249E-97 | -11.37 | hydrogenase-4 component E |
| D7S_02168 | 2.27287E-95 | -11.50 | hydrogenase-4 subunit J |
| D7S_00257 | 5.8526E-124 | -11.58 | putative periplasmic binding protein CbiK |
| D7S_01501 | 1.84128E-64 | -12.15 | fumarate reductase subunit D |
| D7S_01171 | 1.4881E-59 | -12.40 | inner membrane protein YjjP |
| D7S_00104 | 4.2434E-119 | -12.41 | biotin sulfoxide reductase |
| D7S_01502 | 5.66207E-84 | -13.47 | fumarate reductase subunit C |
| D7S_02176 | 3.87956E-49 | -13.51 | hydrogenase-4 component B |
| D7S_00535 | 2.2247E-111 | -14.05 | oxidoreductase domain-containing protein |
| D7S_00030 | 1.3711E-68 | -14.37 | oxaloacetate decarboxylase subunit alpha |
| D7S_02174 | 5.44654E-60 | -16.84 | hydrogenase-4 component D |
| D7S_02167 | 4.46006E-80 | -18.27 | hydrogenase maturation peptidase HycI |
| D7S_00249 | 3.8493E-99 | -18.86 | hydrogenase expression/formation protein HypE |
| D7S_02177 | 3.21706E-78 | -19.82 | electron transport protein HydN |
| D7S_02175 | 3.55639E-56 | -21.50 | hydrogenase-4 subunit C |
| D7S_02172 | 6.596E-146 | -21.71 | hydrogenase-4 component F |
