# Supplementary material for: Transcriptome Profiling of Wild-Type and pga-Knockout Mutant Strains Reveal the Role of Exopolysaccharide in Aggregatibacter actinomycetemcomitans
Source: PLoS One. 2015 Jul 29;10(7):e0134285. doi: 10.1371/journal.pone.0134285 (PMC4519337; doi:10.1371/journal.pone.0134285)
Supplement: S2 Table — EA1002. (DOC) [file pone.0134285.s003.doc]

**S2 Table:. Overrepresented GO terms; Hypergeometric test P-values (<0.05).**

**EA1002**

| **GO Name** | **P Value** |
| --- | --- |
| **Biological process** |  |
| mismatch repair | 0 |
| nucleotide biosynthetic process | 0 |
| fumarate metabolic process | 0 |
| malate metabolic process | 0 |
| porphyrin-containing compound biosynthetic process | 0 |
| ATP synthesis coupled electron transport | 0 |
| peptidoglycan catabolic process | 0 |
| selenocysteine incorporation | 0 |
| 'de novo' AMP biosynthetic process | 0 |
| 1,6-anhydro-N-acetyl-beta-muramic acid catabolic process | 0 |
| SRP-dependent cotranslational protein targeting to membrane | 0 |
| translation | 4.457919E-5 |
| electron transport chain | 0.0003023763 |
| peptidoglycan turnover | 0.001170915 |
| transcription, DNA-templated | 0.001698256 |
| carbohydrate metabolic process | 0.002882036 |
| Mo-molybdopterin cofactor biosynthetic process | 0.004366138 |
| glycolytic process | 0.004366138 |
| cellular amino acid metabolic process | 0.004528356 |
| threonylcarbamoyladenosine biosynthetic process | 0.004528356 |
| aromatic amino acid family biosynthetic process | 0.01517581 |
| chorismate biosynthetic process | 0.01517581 |
| pentose-phosphate shunt | 0.01517581 |
| one-carbon metabolic process | 0.01749018 |
| carbohydrate transport | 0.01749018 |
| N-acetylglucosamine metabolic process | 0.01749018 |
| glycogen biosynthetic process | 0.01749018 |
| nucleotide catabolic process | 0.01749018 |
| response to heat | 0.01749018 |
| L-serine biosynthetic process | 0.01749018 |
| UMP salvage | 0.01749018 |
| CDP-diacylglycerol biosynthetic process | 0.01749018 |
| protein transport by the Tat complex | 0.01749018 |
| FtsZ-dependent cytokinesis | 0.01795812 |
| cell wall organization | 0.02475627 |
| protein folding | 0.0333989 |
| **Molecular function** |  |
| oxaloacetate decarboxylase activity | 0 |
| shikimate 3-dehydrogenase (NADP+) activity | 0 |
| ligase activity | 0 |
| ligase activity, forming carbon-nitrogen bonds | 0 |
| mismatched DNA binding | 0 |
| oxidoreductase activity, acting on NAD(P)H | 0 |
| copper ion binding | 0 |
| N-acetylmuramoyl-L-alanine amidase activity | 0 |
| site-specific DNA-methyltransferase (adenine-specific) activity | 0 |
| C4-dicarboxylate transmembrane transporter activity | 0 |
| glycogen phosphorylase activity | 0 |
| NADH dehydrogenase (ubiquinone) activity | 0 |
| [formate-C-acetyltransferase]-activating enzyme activity | 0 |
| peroxidase activity | 0 |
| structural constituent of ribosome | 1.428714E-6 |
| rRNA binding | 0.0004332668 |
| heme binding | 0.0005177599 |
| aminoacyl-tRNA editing activity | 0.001170915 |
| ATP binding | 0.003107923 |
| NADP binding | 0.003541233 |
| nickel cation binding | 0.004528356 |
| calcium ion binding | 0.004528356 |
| sequence-specific DNA binding transcription factor activity | 0.005260403 |
| lyase activity | 0.01745133 |
| molybdate transmembrane-transporting ATPase activity | 0.01749018 |
| exodeoxyribonuclease V activity | 0.01749018 |
| protein transmembrane transporter activity | 0.01749018 |
| ATP-dependent RNA helicase activity | 0.01749018 |
| double-stranded RNA binding | 0.01749018 |
| electron carrier activity | 0.02753029 |
| amino acid binding | 0.03189364 |
| tRNA binding | 0.03199194 |
| DNA binding | 0.0335523 |
| 4 iron, 4 sulfur cluster binding | 0.03729472 |
| pyridoxal phosphate binding | 0.03729472 |
| 3'-5' exonuclease activity | 0.04284714 |
| ATPase activity, coupled to transmembrane movement of substances | 0.04607415 |
| **Cellular component** |  |
| ribosome | 0.0002635573 |
| cytoplasm | 0.00208395 |
| cell division site | 0.004528356 |
| integral component of membrane | 0.01271249 |
| integral component of plasma membrane | 0.01745133 |
| TAT protein transport complex | 0.01749018 |
