# Supplementary material for: Transcriptome Profiling of Wild-Type and pga-Knockout Mutant Strains Reveal the Role of Exopolysaccharide in Aggregatibacter actinomycetemcomitans
Source: PLoS One. 2015 Jul 29;10(7):e0134285. doi: 10.1371/journal.pone.0134285 (PMC4519337; doi:10.1371/journal.pone.0134285)
Supplement: S3 Table — IDH781. (DOC) [file pone.0134285.s004.doc]

**S3 Table. Overrepresented GO terms; Hypergeometric test P-values (<0.05).**

IDH781

| **GO Name** | **P Value** |
| --- | --- |
| **Biological process** |  |
| carbohydrate biosynthetic process | 0 |
| cellular iron ion homeostasis | 0 |
| iron ion transport | 0 |
| translation | 0 |
| cellular respiration | 0 |
| guanosine tetraphosphate metabolic process | 0 |
| glutamine metabolic process | 0.003394662 |
| mRNA catabolic process | 0.003394662 |
| tRNA processing | 0.008291765 |
| cell morphogenesis | 0.01409357 |
| regulation of translation | 0.01409357 |
| cell wall macromolecule catabolic process | 0.01409357 |
| peptidoglycan metabolic process | 0.01409357 |
| rRNA base methylation | 0.01409357 |
| barrier septum assembly | 0.02351866 |
| lipid A biosynthetic process | 0.02351866 |
| DNA replication | 0.03092639 |
| queuosine biosynthetic process | 0.0332851 |
| ribosome biogenesis | 0.0461903 |
| **Molecular function** |  |
| rRNA binding | 0 |
| large ribosomal subunit rRNA binding | 0 |
| structural constituent of ribosome | 0 |
| tRNA binding | 0.0001541918 |
| ATP binding | 0.001617645 |
| ferric iron binding | 0.003394662 |
| translation initiation factor activity | 0.003394662 |
| protein disulfide oxidoreductase activity | 0.008291765 |
| formate dehydrogenase (NAD+) activity | 0.01370733 |
| nucleic acid binding | 0.01384664 |
| lytic transglycosylase activity | 0.01409357 |
| ribosome binding | 0.01409357 |
| amino acid binding | 0.02351866 |
| hydrolase activity, hydrolyzing O-glycosyl compounds | 0.0332851 |
| receptor activity | 0.04255227 |
| structural molecule activity | 0.0461903 |
| sigma factor activity | 0.0461903 |
| aromatic amino acid transmembrane transporter activity | 0.0461903 |
| oxidoreductase activity, acting on the CH-OH group of donors, NAD or NADP as acceptor | 0.0461903 |
| **Cellular component** |  |
| large ribosomal subunit | 0 |
| ribosome | 6.661338E-16 |
| small ribosomal subunit | 4.703367E-5 |
| proton-transporting ATP synthase complex, coupling factor F(o) | 0.01409357 |
| ATP-binding cassette (ABC) transporter complex | 0.02851612 |
